# Imaging Saturation Transfer Difference (STD) NMR: Affinity and Specificity of Protein–Ligand Interactions from a Single NMR Sample

**DOI:** 10.1021/jacs.3c02218

**Published:** 2023-07-24

**Authors:** Serena Monaco, Jesus Angulo, Matthew Wallace

**Affiliations:** † School of Pharmacy, University of East Anglia, Norwich Research Park, Norwich NR4 7TJ, U.K.; ‡ Instituto de Investigaciones Químicas (IIQ), Consejo Superior de Investigaciones Científicas and Universidad de Sevilla, Avenida Américo Vespucio, 49, Sevilla 41092, Spain

## Abstract

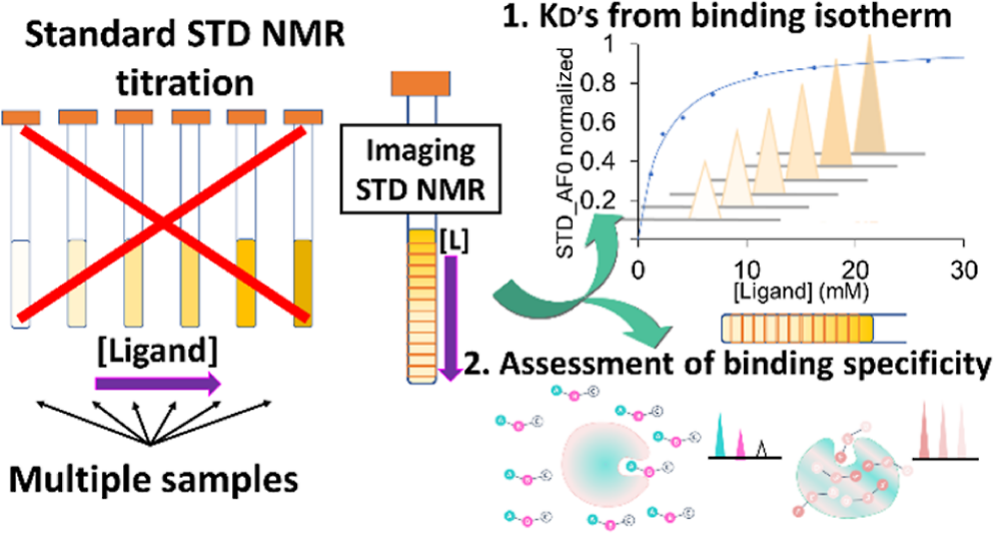

We have combined saturation transfer difference NMR (STD NMR) with chemical shift imaging (CSI) and controlled concentration gradients of small molecule ligands to develop imaging STD NMR, a new tool for the assessment of protein–ligand interactions. Our methodology allows the determination of protein–ligand dissociation constants (*K*_D_) and assessment of the binding specificity in a single NMR tube, avoiding time-consuming titrations. We demonstrate the formation of suitable and reproducible concentration gradients of ligand along the vertical axis of the tube, against homogeneous protein concentration, and present a CSI pulse sequence for the acquisition of STD NMR experiments at different positions along the sample tube. Compared to the conventional methodology in which the [ligand]/[protein] ratio is increased manually, we can perform STD NMR experiments at a greater number of ratios and construct binding epitopes in a fraction (∼20%) of the experimental time. Second, imaging STD NMR also allows us to screen for non-specific binders, by monitoring any variation of the binding epitope map at increasing [ligand]/[protein] ratios. Hence, the proposed method does carry the potential to speed up and smooth out the drug discovery process.

## Introduction

Investigating the nature of the molecular recognition processes between biomolecules allows us to understand how their specific interactions regulate essential processes of life. This is of paramount importance both in drug design and fundamental biological investigations. NMR is among the election techniques to carry such investigation, and saturation transfer difference NMR (STD NMR), among other ligand-based NMR techniques, stands out as a reliable and easy to apply ligand-based methodology which can provide invaluable insights into the mode of binding (binding epitope mapping) and strength of the interaction (dissociation constant, *K*_D_) of protein–ligand complexes on the weak affinity range (μM to mM).^1,2^

In this work, we combine STD NMR with chemical shift imaging (CSI) NMR to develop imaging STD NMR. The CSI experiments use gradient phase encoding to acquire 1D ^1^H STD NMR spectra at each depth, or slice, of the protein sample along a concentration gradient of the ligand in a single experiment.^3^ The CSI approach contrasts with the slice-selection approach in that spectra are obtained simultaneously at regular intervals along the sample, rather than from a single selected region.^4^ Here, each slice represents one [ligand]/[protein] ratio ([L]_T_/[P]_T_, with the T subscript indicating total molar concentration), “replacing” one sample of the traditional titration approach in which small additions of the ligand stock are manually added to the tube to gradually increase the [ligand]/[protein] ratio. The implementation of imaging STD NMR that we propose here enables us to determine dissociation constants (*K*_D_), and assess binding specificity, in a single sample tube. Unlike other published methods for the single-sample determination of *K*_D_ that are based on the measurement of the line width of the ligand, our STD-based approach requires fewer assumptions about the nature of the protein–ligand complex and relaxation rates and so can be applied to a greater range of systems with confidence.^5^ The application of CSI NMR to a sample containing a concentration gradient has been proposed^6^ as a way to condense, in a single-tube, experiments traditionally requiring titrations, i.e., preparation of separate samples at variable concentrations of a given component. This approach has already been applied to determine the p*K*_a_ and p*K*_b_ of small molecules in aqueous and non-aqueous solvents,^7^ the study of weak molecular interactions through concentration gradients in agar gels,^8^ reaction monitoring,^9^ and binding affinity of macromolecules to Ca^2+^ and Mg^2+^.^10^

**Figure 1:**
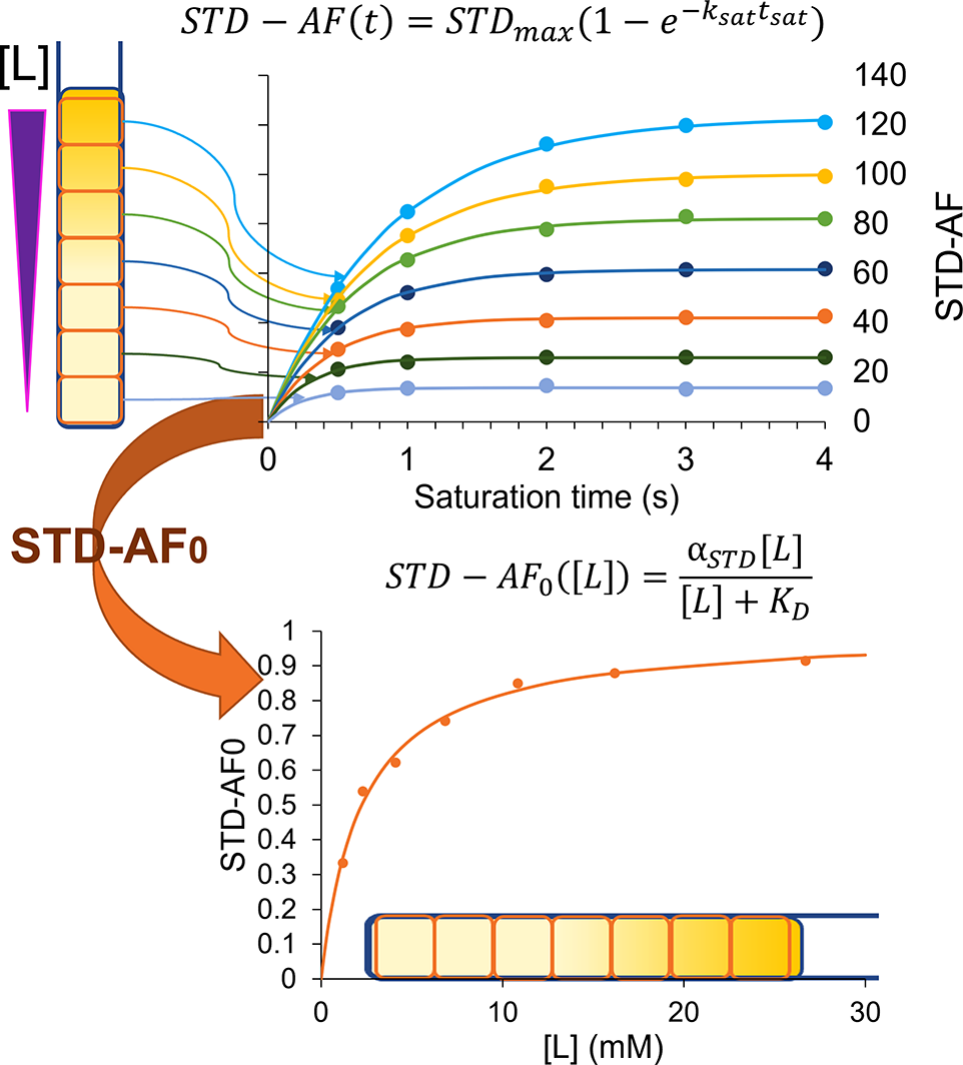
Sketch of the Imaging STD NMR approach for the determination of dissociation constants based on initial growth of the build-up curves. An STD-NMR build-up curve is extracted at each depth of the tube, corresponding to increasing [ligand]/[protein] ratios (bottom to top). From these, the initial slopes are calculated and plotted against the ligand concentration to obtain the binding isotherm and hence *K*_D_.

STD NMR relies on the homogeneous saturation of the receptor (generally a protein), which allows magnetization transfer to any bound ligand. For each signal, the transferred saturation, reflected by the STD factor [η_STD_, defined as (*I*_0_ – *I*_sat_)/*I*_0_], is correlated to the proximity of the given set of protons to the protein surface, which translates into the binding epitope map of the ligand in the complex.^11^ The η_STD_ multiplied by the excess of ligand, [L]_T_/[P]_T_, is called the STD amplification factor (STD-AF) and is proportional to the concentration of the protein–ligand complex in solution. Plotting STD-AF against the ligand concentration, [L]_T_, we can therefore obtain binding isotherms for the complexes and hence obtain the apparent *K*_D_’s of the complexes.^12,13^ The saturation time, *t*_sat_, at which STD-AFs are recorded, i.e., the time for which the presaturation is applied to the protein, determines the accuracy of *K*_D_ determination as the effect of ligand rebinding increases at longer saturation times.^14^ To avoid this effect, it is necessary to acquire STD build-up curves, recording STD-AF as a function of saturation time, at increasing [L]_T_/[P]_T_ ratios, as sketched in Figure 1. K_D_’s can then be extracted from binding isotherms built with initial growth rates of STD-AF (STD-AF_0_).^14^ All the equations and mathematical definitions are reported in Section S2 of the Supporting Information.

Although efficient in measuring accurate *K*_D_ values, the STD NMR approach is particularly dispendious as it requires a number of manual ligand additions to the samples equal to the desired number of [L]_T_/[P]_T_ ratios. Acquisition of full build-up curves after each addition also limits the number of ratios that can be assessed, if time and resources are limited or experimental conditions do not allow (for example, if the protein is particularly unstable in solution). In contrast, as summarized in Figure 1, imaging STD NMR allows us to measure dissociation constants in a single NMR sample constituted of a gradient of ligand developing along the vertical axis, against a homogeneous receptor concentration.

Additionally, using the same method, we can assess binding specificity of a given molecule to a protein target through the variation of its binding epitope with ligand concentration. The binding epitope is a map of how the STD response is distributed around the molecule, and thus which protons are closest to those of the protein.^1,11^ Cala and Krimm have shown that assessing the binding epitope map at increasing [ligand]/[protein] ratio is an efficient tool to discriminate between specific and non-specific binding to the protein.^15^ The binding epitope map of specific binders is independent of ligand concentration, while non-specific binders lose their binding epitope map information at increasing ligand concentration because of their binding all over the protein surface with multiple non-repetitive orientations, resulting in the complete loss of binding epitope information. If undetected, non-specific binders can be mistaken for promising leads and brought further in the drug discovery process, invalidating it.^16^ It is therefore urgent to reveal and exclude non-specific binders at an early stage. As the pool of techniques apt to spot non-specific binders is limited, we test the potential of imaging STD NMR to assess the specificity of binding, along with the measurement of *K*_D_, in a one-shot single tube experiment.

## Results and Discussion

### Creating Controlled Ligand Gradients

**Figure 2:**
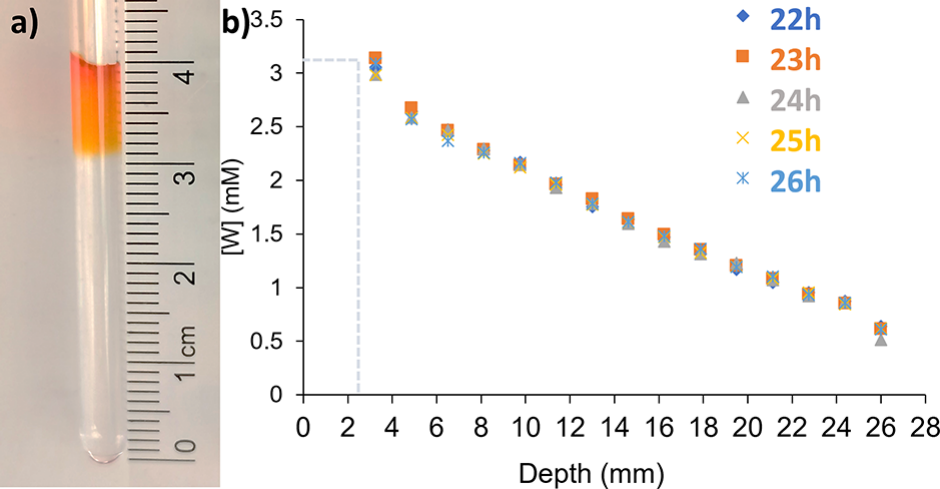
(a) Example concentration gradient produced by placing 50 μL of methyl orange solution on top of 400 μL of BSA protein in buffer. (b) Diffusion profile of tryptophan between 22 and 26 h, showing the stability of the concentration gradient needed for analysis by imaging STD NMR. Depth is the distance from the boundary, where the tryptophan solution was layered (31 mm from the tube bottom).

To create controlled and reproducible gradients along the vertical axis of the 5 mm NMR tube (as shown in Figure 2a), we approximate the tube to an infinite cylinder at the top of which a given amount of solute is placed and allowed to diffuse.^17^ The concentration, *C*, of the solute at each height *z* from the top of the tube is given by eq 1
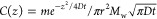
1where *m* is the total mass of solute, *r* is the inner radius of the NMR tube (2.1 mm), *M*_W_ is the molecular weight of the solute, *D* is the diffusion coefficient, and *t* is the time elapsed since preparation of the sample. We have previously shown that a solid solute can be placed on the bottom of the tube, covered by glass beads, and a solution placed on top to create a stable and reproduceable concentration gradient by dissolution and diffusion.^6,18^ This approach works well for non-sticky solids like acids or salts. However, it is more challenging for sticky hydrophobic organic solids which tend to smear along the NMR tube. Furthermore, minuscule quantities of the solid would be required to determine small *K*_D_ values that would be unfeasible to weigh out. Therefore, we decided to use a concentrated aqueous solution of the solute as the source for diffusion in the homogeneous protein solution.

Protein solutions, due to their high buffer concentration, are generally denser than concentrated solutions of organic ligands. We therefore diffuse the ligand from top to bottom, carefully placing a solution of ligand on top of the protein solution, as shown in Figure 2a. In Section S11 of the Supporting Information, we also show that, should ligand solubility be a problem, heavier dimethyl sulfoxide (DMSO)-containing ligand stocks can be placed at the bottom of the tube, without affecting the development and predictability of the gradients.

Rearranging eq 1, we obtain
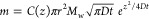
2

To calculate the exact mass required to achieve the desired concentration window, we first need to determine the diffusion coefficient *D*. By creating a ligand gradient in the absence of protein at known diffusant volume and concentration at a given time, *D* can be calibrated for each ligand so that the experimental concentration 4 mm below the boundary of the ligand and protein solutions (*z* = 4 mm, Figure 2a) matches the prediction from eq 1. The diffusion coefficients experimentally determined for tryptophan, *N*-acetylglucosamine (GlcNAc), and 3-nitrophenyl-α-d-galactopyranoside (3NPG) with this approach were 5 × 10^–10^, 4 × 10^–10^, and 3 × 10^–10^ m^2^/s, respectively. These values are in good agreement with the predictions obtained using the approach presented by Evans et al.,^19^ which gave the values of 5.29 × 10^–10^, 5.09 × 10^–10^, and 3.83 × 10^–10^ m^2^/s, respectively, for tryptophan, GlcNAc, and 3NPG, so predictions based on the molecular weight of the ligand could be an alternative viable approach.

Once *D* is obtained for a ligand, we can fix the required maximum concentration at the top of the tube (for example, at 4 mm from the top of the boundary as this is the first slice that we can read), at a given time. Based on the mass obtained from eq 2, the exact volume and concentration required are calculated, aiming at volumes between 20 and 50 μL that can be easily pipetted down the wall of the tube.

An example of this approach is reported in Figure 2b, where we aimed for a maximum concentration of 3 mM tryptophan 4 mm from the top of the boundary, 22 h after preparation. Tryptophan (23 μL, 30 mM) was then layered on top of 400 μL of buffer solution, as calculated according to eq 2, and the gradient left to develop for 22 h before the spectra were acquired. The concentration of the ligand is determined by integration against the internal NMR standard, in our case pyrazine. As highlighted by the gray dotted lines, the observed value at these fixed conditions exactly matches the calculations. Importantly, this same figure shows that the gradient is stable over 4 h between 22 and 26 h. This is supported by the diffusion profile simulations reported in Figure S6 of the Supporting Information, showing how for small molecular weight ligands, concentration gradients become more stable 18 h after preparation, with 22–26 h being a suitable time for the analysis. The gradient stability enables the acquisition of build-up curves as a train of experiments at increasing saturation times, so as to ensure the accurate measurements of *K*_D_. Total experimental time depends on the number of saturation times measured and the concentration of the ligand and number of scans required, but 2 h 30 min was sufficient for the acquisition of build-up curves for the tryptophan/bovine serum albumin system, where the concentration ranged between 0.3 and 1.3 mM. The sample needs no adjustment during this period, and the required series of STD experiments can be recorded automatically. Our method thus avoids experimental complications related to the incomplete mixing or increase in volume of the sample over the course of a titration as a ligand is added.

### *K*_D_ Determination in a Single Tube

**Figure 3:**
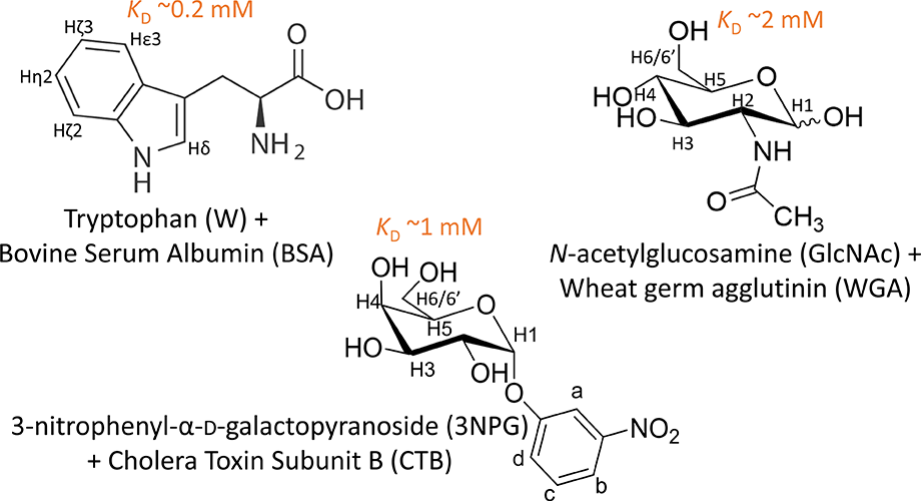
Structure and atom nomenclature of the ligands in the protein–ligand complexes used as model systems in this study.

As model systems, we used: (i) tryptophan (W) bound to bovine serum albumin (BSA), (ii) GlcNAc bound to wheat germ agglutinin (WGA), and (iii) 3NPG as bound to cholera toxin subunit B (CTB). Ligands are shown in Figure 3. For these three complexes, the dissociation constants are reported in the literature, ranging from 200 μM (in the lower region of the affinity window detectable by STD NMR) for W/BSA, to 2 mM for the GlcNAc·WGA, while 3NPG/CTB is in the middle with a *K*_D_ of about 1 mM. Additionally, *W* exhibits non-specific binding to BSA due to the intrinsic lipophilicity of albumins. In contrast, GlcNAc/WGA and 3NPG/CTB show very specific binding as it is often the case for carbohydrate recognizing domains.^20,21^

For each system, on the sample containing the ligand gradient against the homogeneous concentration of protein, we acquired a set of STD NMR build-up curves using Imaging STD NMR. This was achieved by adding pre-saturation to ^1^H CSI pulse sequence,^3^ incorporating perfect echo water suppression block to be able to analyze samples at different light water contents.

**Figure 4:**
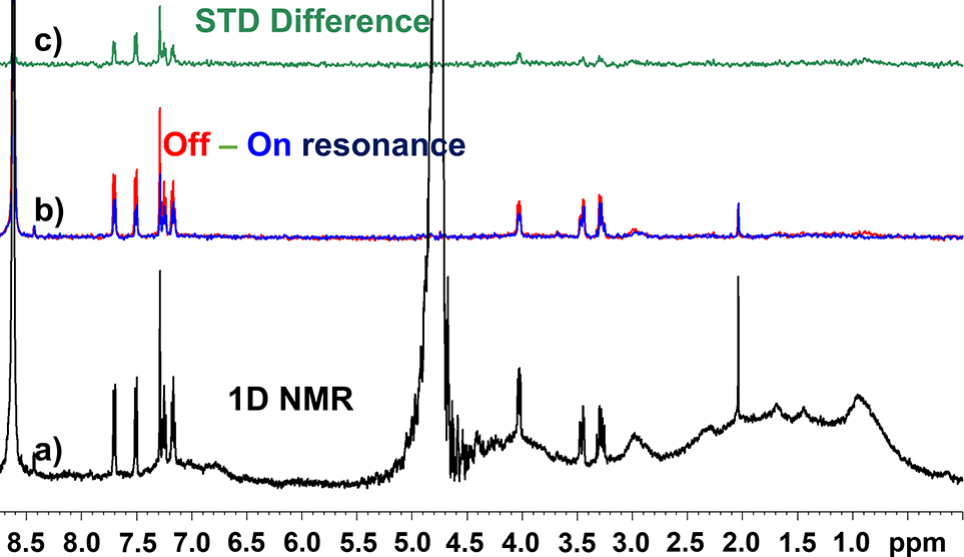
Spectra from imaging STD NMR experiments of a sample containing a gradient of tryptophan against homogeneous concentrations of BSA. (a) 1D NMR spectrum of the sample acquired with a 30° pulse, without water suppression. (b) On- and off-resonance spectra and (c) STD difference spectra of slice 8 of 16 of the imaging STD experiment performed on the same sample. The imaging STD NMR experiment was acquired with eight scans for an experimental time of 22 min.

**Figure 5:**
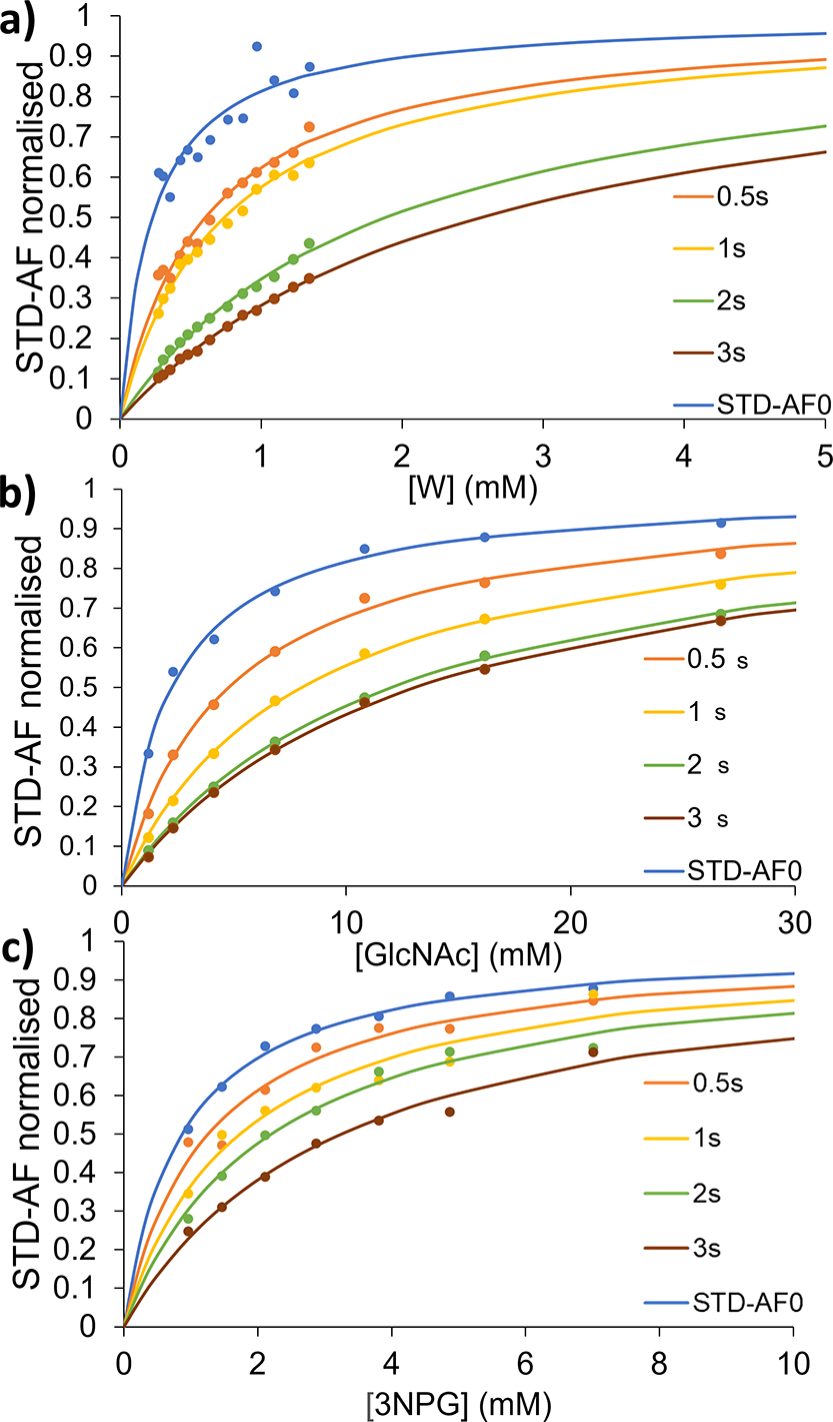
Imaging STD NMR Langmuir binding isotherms for *K*_D_ determination of the (a) tryptophan/BSA complex, based on the average of all the aromatic protons; (b) and GlcNAc/WGA, based on the methyl group signal; and (c) 3NPG/CTB complex, based on the H2,3,5 proton signal of the sugar ring. For the three complexes, we show the binding isotherms (lines) obtained from fitting either the initial slopes of build-up curves (STD-AF0), in blue dots, or from the STD-AF at increasing saturation time, in orange to brown dots. Tabulated data for the binding isotherms are reported in Section S4 of the Supporting Information, where the STD NMR build-up curves obtained at each depth of the tube, i.e., at increasing ligand concentration, and tabulated data, are also included.

Figure 4 shows an example of the resulting spectra, and for more details, we send the reader to Sections S1 (Experimental section), S3 (imaging STD NMR control experiments and sensitivity assessment) and S9 (pulse sequence for the STD CSI experiment) of the Supporting Information. For the three systems, the binding isotherms obtained from different saturation times and from STD-AF_0_ are reported in Figure 5.

*K*_D_ values were extracted from all the binding isotherms, both from STD-AF initial growth rates (STD-AF_0_) and from STD-AF measured at individual saturation times. Excellent fits to the Langmuir equation (eq S6) were obtained in all cases (*R*^2^ > 0.99). However, in cases where high ligand excess cannot be ensured, an alternative fitting equation should be used that explicitly considers the total concentrations of protein and ligand. Both equations give equivalent results in the present work (Section S5 of the Supporting Information). In case a full STD build-up analysis cannot be carried out (e.g., limitations in protein availability and/or stability), a single saturation time titration experiment will provide an upper limit of the dissociation constant. In those cases, to get the best *K*_D_ approximation, low saturation times and low protein concentrations should be used to minimize underestimation of affinity due to ligand rebinding^14^ (for a discussion and comparison of the *K*_D_ obtained from STD-AF_0_ and from single saturation times, see Section S6 of the Supporting Information). The *K*_D_ data obtained for the three systems from imaging STD NMR are reported in Table 1, in comparison with the conventional STD NMR titration reported in the study by Angulo et al.^14^ and isothermal titration calorimetry (ITC) or weak affinity chromatography (WAC) measurements reported in the literature.

**Table 1:** Comparison of Dissociation Constants Obtained by Imaging STD NMR, Traditional STD NMR Titration and ITC or WAC, and the Number of [L]_T_/[P]_T_ Ratios Investigated and Instrument Time

	imaging STD NMR	STD NMR titration^14^	ITC or WAC
	W/BSA		
*K*_D_	231 ± 50 μM	190 ± 20 μM	230 ± 90 μM^22^
number of [L]_T_/[P]_T_	13	5	
instrument time^a^	2 h 30 min	30 h	
	GlcNAc/WGA Lectin		
*K*_D_	2.24 ± 0.18 mM	2.4 ± 0.3 mM	2.5 ± 0.15 mM^20^
number of [L]_T_/[P]_T_	7	7	
instrument time^a^	2 h	21 h	
	3NPG/CTB		
_D_	0.93 ± 0.7 mM		1.1 ± 0.1 mM^23^
number of [L]_T_/[P]_T_	7		
instrument time^a^	2 h 45 min		

a Instrument time for the STD NMR titration is calculated as the sum of the instrument time required to obtain each *K*_D_ in our work and as reported in ref (14), accounting for the original conditions, so as to allow direct comparison of the values obtained here and in that work.

The *K*_D_’s obtained by imaging STD NMR are comparable to those reported in the literature within the experimental uncertainties. For the W/BSA system, the uncertainty in *K*_D_ is larger than the error obtained from the STD NMR titration (but smaller than error coming from ITC). This can be ascribed by the low *K*_D_ (0.2 mM), requiring low concentrations, and thus higher uncertainty in the concentration of ligand determined by integration. The uncertainty associated with the *K*_D_ measurement for the GlcNAc/WGA complex is smaller relative to the STD NMR titration and comparable to ITC measurements as the higher *K*_D_ and ligand concentration and the presence of a strong methyl signal allow for more accurate integration.

We note that imaging STD NMR requires only 20% of the experimental time required for conventional STD NMR titrations and readily allows us to analyze up to 14 [L]_T_/[P]_T_ ratios, whereas acquiring more than five points is logistically challenging with the original method, due to many factors, including protein stability over the long run. Also, for mM concentrations, our methodology may be more accurate than manual titration where the sample is repeatedly manipulated, and human factors have a larger impact. Further details on comparison between our methodology and manual STD NMR titration for *K*_D_ determination are provided in Section S7 of the Supporting Information, while the effect of the protein concentration is discussed in Section S12.

### Binding Specificity Assessment in a Single Tube

The second application of imaging STD NMR is the assessment of binding specificity in a single tube. It is important to remark that for this application, a new gradient sample is normally required as the concentration window required for specificity analysis is larger than that required for the *K*_D_ measurements. Ideally, measurements are performed at concentrations up to at least 10× *K*_D_, to evaluate the evolution of the binding epitope mappings upon increasing the excess of ligands.

**Figure 6:**
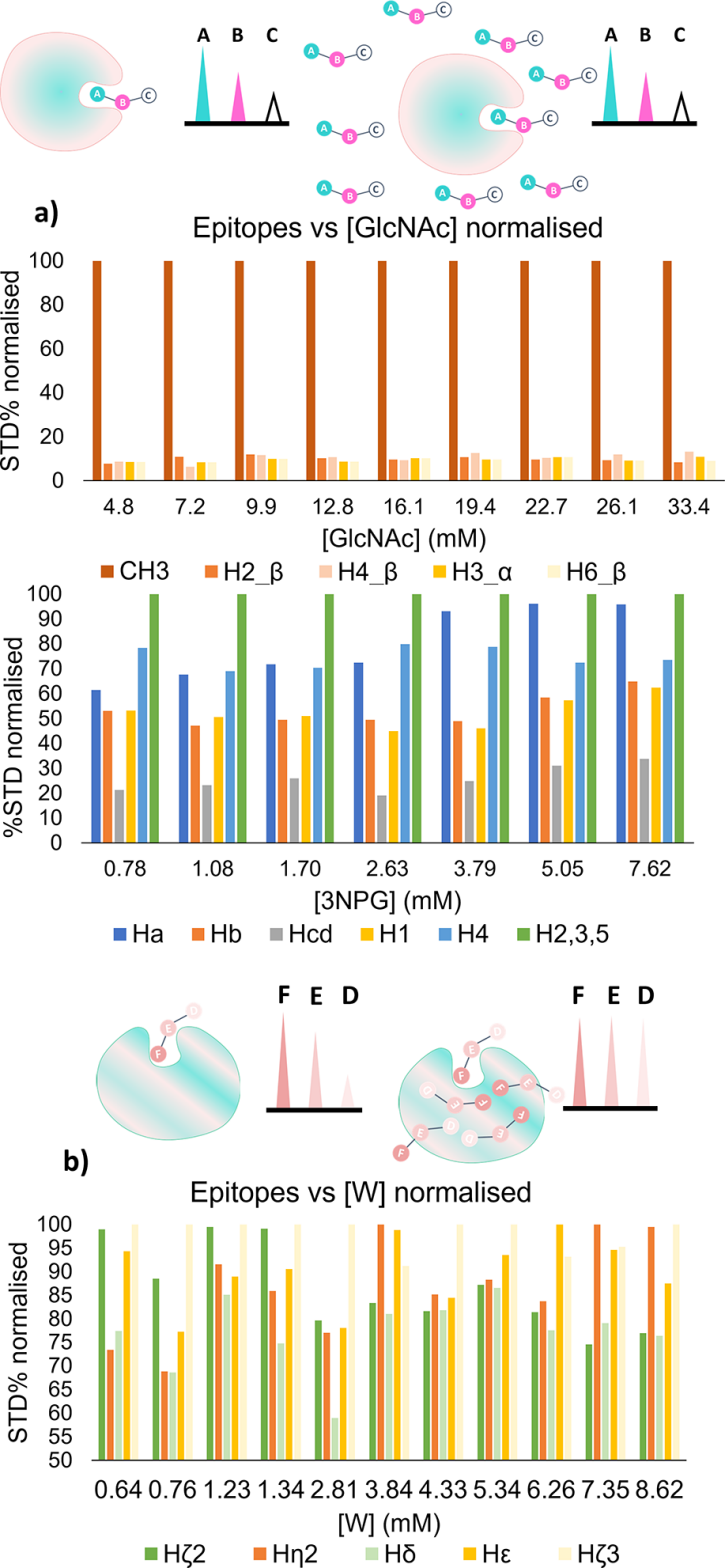
Assessment of binding specificity by imaging STD NMR. Top: cartoons of how STD NMR binding epitopes can be used for assessing the specificity of binding, where a specific protein–ligand complex is represented in (a), and a non-specific protein–ligand complex is represented in (b). Bottom: histograms of the binding epitope mapping of the complexes GlcNAc/WGA (top) and W/BSA (bottom) obtained from initial slopes derived from imaging STD NMR build-up curves at increasing ligand concentration, from a single tube. For the atom nomenclature, see Figure 3. GlcNAc-binding epitopes are normalized to the methyl group which gave the strongest STD response. The strongest STD response exhibited by the tryptophan changed for each concentration due to non-specific binding. Tabulated data are reported in Section S8.

As shown in Figure 6, the two different scenarios of a specific and non-specific protein–ligand complex are sketched. For a specific binder, even large ligand excess will not affect the binding epitope as once the binding site is saturated, there is no chance for the ligand to bind anywhere else. For non-specific binders, once the binding site is saturated, the ligand can bind everywhere else on the protein surface, resulting in loss of binding epitope mapping information. Below the sketches, the experimental normalized binding epitope mappings at increasing ligand concentration are reported for GlcNAc/WGA and 3NPG/CTB, and W/BSA. It is evident, from the comparison of the two histograms, that GlcNAc and 3NPG exhibit specific binding to the WGA lectin and CTB-binding domain (Figure 6a) as the binding epitope remains constant at increasing ligand concentration. Once the binding site is saturated, the excess ligand stays free in solution without any non-specific interaction with the protein surface. On the contrary, for the W/BSA complex, we can initially see a binding epitope pattern with Hζ2 and Hζ3 receiving higher saturation relative to Hε, η2, and δ.

This epitope pattern is completely lost when the ligand concentration goes above 1–2 mM as once the binding site is saturated, tryptophan starts interacting in a non-specific manner with the (very lipophilic) BSA protein surface (Figure 6b). While Cala and Krimm propose the study of the binding epitope at two different [ligand]/[protein] ratios to test for specificity,^15^ our imaging STD NMR experiment is potentially more reliable due to the higher number of ratios investigated.

## Conclusions

We have hereby developed imaging STD NMR, by combining CSI NMR and STD NMR, to obtain spatially resolved STD NMR experiments along the *z*-axis of an NMR tube and apply it to samples containing ligand gradients against a homogeneous protein concentration. We have implemented this methodology into two distinguished applications, the first allowing us to determine dissociation constants in a single NMR tube and the second allowing us to assess the specificity of binding. We consider the application of imaging STD NMR to assess the specificity of binding an important implementation as detecting non-specific binder is a challenging task, and a limited number of methodologies are available to the scope.

Finally, we envision an even wider applicability of imaging STD NMR, going beyond the *K*_D_ determination and specificity of binding assessment. This could range from the study of protein–ligand interactions in the presence of gradients of competitors, as well as at variable pH values, and various co-solvent concentrations to identify optimal conditions for binding. Furthermore, beyond the realm of protein–ligand complexes, the same qualitative approach could be used to structurally characterize key molecular interactions in a broad range of systems such as polymer emulsions,^24,25^ micellar pharmaceutics,^26^ and DNA-based molecular machines, at variable conditions in a single tube.

## Data Availability

Research data will be available at: https://people.uea.ac.uk/en/datasets/.

## Supporting Information Available

Experimental section; mathematical derivation of STD-AF_0_ and of *K*_D_ from the STD-AF_0_-binding isotherm; imaging STD NMR control experiments and comparison of STD NMR and imaging NMR spectra; build-up curves with data and binding isotherms data for the three complexes; mathematical derivation of more general fitting for *K*_D_ determination; *K*_D_ values at single saturation times; comparison for instrument time for STD NMR titration and imaging STD NMR; binding epitope mappings at increasing concentrations for W/BSA and GlcNAc/WGA; pulse sequence for the STD CSI experiment (Bruker); amount of mass and concentration for gradient formation of small molecules; effect of DMSO in the stock of the diffusion profile; effect of the protein concentration on sensitivity and simulation of protein diffusion; macros for automated imaging STD NMR data processing (Bruker); STD NMR data processing: manual method and automation; and STD NMR data processing on Mnova 14.3.1 by line fitting (PDF)
Calculation of mass and concentration of ligands and for imaging STD NMR data processing (XLSX)

## Abbreviations

STD NMR: saturation transfer difference NMR
CSI: chemical shift imaging
STD-AF: STD amplification factor
W: tryptophan
BSA: bovine serum albumin
GlcNAc: *N*-acetylglucosamine
WGA: wheat germ agglutinin
3NPG: 3-nitrophenyl-α-*d*-galactopyranoside
CTB: cholera toxin subunit B
ITC: isothermal titration calorimetry
WAC: weak affinity chromatography
