# Supplementary material for: Imaging Saturation Transfer Difference (STD) NMR: Affinity and Specificity of Protein–Ligand Interactions from a Single NMR Sample
Source: J Am Chem Soc. 2023 Jul 24;145(30):16391–7. doi: 10.1021/jacs.3c02218 (PMC10401705; doi:10.1021/jacs.3c02218)
Supplement: Supplementary file 1 — ja3c02218_si_001.pdf [file ja3c02218_si_001.pdf]

Supporting Information - Imaging Saturation Transfer Difference (STD) NMR: affinity and specificity of protein-ligand interactions from a single NMR sample

Serena Monaco[a], Jesus Angulo[b], Matthew Wallace[a]

s.monaco@uea.ac.uk, matthew.wallace@uea.ac.uk

a. School of Pharmacy, University of East Anglia, Norwich Research Park, Norwich, NR4 7TJ, UK
b. Instituto de Investigaciones Químicas (IIQ), Consejo Superior de Investigaciones Científicas and Universidad de Sevilla, Avenida Américo Vespucio, 49, Sevilla, 41092, Spain

Contents

S1. Experimental section 2

S2. Mathematical derivation of STD-AF$_0$ and of K_D from the STD-AF$_0$ binding isotherm 3

S3. Imaging STD NMR control experiments and comparison of STD NMR and Imaging STD NMR spectra 4

S4. Build-up curves with tabulated data and binding isotherms tabulated data for the three complexes 7

S5. Mathematical derivation of more general fitting for K_D determination 9

S6. Tables of K_Ds at single saturation times 10

S7. Table of comparison for instrument time for STD NMR titration and Imaging STD NMR 10

S8. Binding epitope mappings at increasing concentrations for W/BSA and GlcNAc/WGA 12

S9. Pulse sequence for STD CSI experiment (Bruker) 14

S10. Excel spreadsheet for amount of mass and concentration for gradient formation of small molecules 18

S11. Effect of DMSO in the stock of diffusion profile 19

S12. Effect of protein concentration on sensitivity and simulation of protein diffusion 20

S13. Macros for automated Imaging STD NMR data processing (Bruker) 22

S13.1 Macro for automatic phasing, baseline correction and referencing. 22

S13.2 Macro for automatically producing difference spectra for each slice 24

S13.3 Macro for STD factor calculation of each STD NMR difference slice 25

S13.4 Macro for running Imaging STD NMR on Icon 26

S14. STD NMR data processing: manual method and automation 28

S15. STD NMR data processing on Mnova 14.3.1 by line fitting 30

S1. Experimental section

Deuterium oxide (99.8%), tryptophan (W), *N*-acetylglucosamine (GlcNAc), pyrazine, Bovine Serum Albumin (BSA, EC number: 232-936-2, purity ≥98%), Wheat Germ Agglutinin (WGA or Lectin from *Triticum vulgaris*, product number 61767), Cholera Toxin Subunit B (CTB, product number C9903, purity ≥95%) and all other buffer reagents were purchased from Sigma-Aldrich. 3-nitrophenyl-α-D-galactopyranoside (3NPG) was purchased from Biosynth Ltd. All the reagents were used as received.

Sample preparation. All experiments were performed in 5 mm Wilmad 528-PP NMR tubes (inner radius 2.1 mm), in D_2O. Protein-ligand gradient samples were prepared by placing homogeneous solutions containing PBS buffer, protein, and pyrazine at the bottom of the NMR tube, spinning the solution down on a Hettich 1011 hand-wound centrifuge to remove any residual protein from the walls of the tube. Thereafter, a small amount (ranging between 20 and 50 µL) of concentrated ligand stock solution, calculated according to Equation (2) in the main text, was pipetted down the walls of the tube and briefly spun down to make sure the entire volume reached the interface with the protein solution. The sample was then left to develop at room temperature for the desired amount of time. Protein solutions were prepared in 137 mM NaCl, 10 mM Na_2HPO_4 and 1 mM KH_2PO_4 adjusted at pH 7.2, uncorrected for D_2O, as measured by a Fisherbrand Hydrus 300 pH meter for both proteins. In the buffer for WGA, 50 mM KCl was added, while buffer for BSA and CTB did not contain any KCl. Pyrazine was always present at 10 mM in the protein stock solutions. The concentrations of pyrazine and protein used in calculations of K_D were corrected for the small volume of ligand added.

All Imaging STD NMR samples were planned by following the calculations made using Equation (2) in the main text, by setting the desired concentration at the top of the tube (4 mm). Initially, 20 µL were placed on top of 480 µL of homogeneous protein solution and an Imaging STD NMR experiment was performed after 18h. Using the known mass of ligand and known concentration at the top of the tube, D was calibrated, and the diffusion coefficients found for tryptophan, N-acetylglucosamine and 3-nitrophenyl-α-D-galactopyranoside were $5x10^{-10}$ m^2/s, $4x10^{-10}$ m^2/s and $3x10^{-10}$ m^2/s, respectively. With these values, the equation was then reverted, and the exact mass required to achieve the desired ligand concentration at the top of the tube (upper concentration limit) was obtained.

For K_D determination, we aimed at 1.5 mM max tryptophan concentration, 20 mM max GlcNAc concentration and 5 mM max 3NPG concentration (at 4 mm, after respectively 18 h, 12 h and 21 h). The position at 4 mm was chosen as each of the 16 slides is 1.625 mm long and the first 1 or 2 slices in proximity of the boundary must be excluded from the analysis due to off-coil effects. The sample composition was: W/BSA sample = 490 µL BSA 50 µM, 10 mM pyrazine, with 9 µL 37.5 mM tryptophan placed on top; GlcNAc/WGA = 450 µL WGA 56 µM, 10 mM pyrazine, with 50 µL 50 mM GlcNAc placed on top; 3NPG/CTB sample = 450 µL CTB 22 µM binding sites (5.5 µM protein concentration, but CTB has 5 identical binding sites), with 30 µL 18 mM 3NPG placed on top.

For binding specificity assessment, we aimed for a maximum concentration of 10 mM tryptophan and 35 mM GlcNAc (at 4 mm, after respectively 18 h and 12 h). The sample composition was therefore as follows: W/BSA sample (450 µL) BSA 50 µM, 10 mM pyrazine, with 50 µL 37.5 mM tryptophan placed on top; GlcNAc/WGA (450 µL) WGA 56 µM, 10 mM pyrazine with 50 µL 80 mM GlcNAc placed on top. For 3NPG/CTB, the same sample was used for both K_D determination and binding specificity assessment.

NMR analysis. All Imaging STD NMR experiments were performed on a Bruker AVANCE NEO 500 MHz spectrometer fitted with a 5mm broadband z-gradient iProbe.

Imaging STD NMR experiments were performed using a gradient phase encoding sequence based on that of Trigo-Mouriño *et al.*, [1] and incorporating the excitation sculpting pulsed gradient perfect echo (Bruker library ZGESGPPE [2]) to suppress the residual HDO resonance and unwanted protein signal, acting as a T_2 filter to suppress the protein resonances. Saturation was delivered using a train of selective 180 degree pulses (Eburp2.1000, 40 ms) prior to the initial hard 90 degree pulse. A spoil gradient (27 G/cm) was employed at the end of the signal acquisition period to destroy any transverse magnetization. The gradient pulse was 84.491 µs in duration and varied between -9.6 G/cm to +9.6 G/cm in 16 steps. The shape of the pulse was a smoothed square. At each step, 8 scans were acquired with the pre-saturation applied at 0.87 ppm, followed by 8 scans with the pre-saturation applied at 40 ppm (off resonance), giving a total acquisition time of 20 min and a theoretical spatial resolution of 1.6 mm. 4 dummy scans were acquired prior to signal acquisition. To obtain STD NMR build-up curves at each increment, a train of Imaging STD NMR experiments were launched with saturation time increasing between 0.5 s and 3 s for W/BSA and 3NPG/CTB and 4 s for GlcNAc/WGA. For all the experiments, the overall relaxation delay, including presaturation pulses, was set to 3 s or 4 s, respectively for W/BSA or 3NPG/CTB and GlcNAc/WGA. The lowest saturation time experiment was acquired with 16 or 32 scans to improve signal-to-noise ratio at low STD intensities.

Data processing. Initial step of the analysis is the determination of ligand concentration $[L]_T$ at each increment of the tube. This is done by the integral ratio between the analyte protons and the internal standard at known concentration, adjusted for the volume of ligand solution added, on the reference (off-resonance) spectra. The ligand concentration is given by the ratio of the absolute integral of ligand over the absolute integral of pyrazine, multiplied by the total concentration of pyrazine protons and divided by the number of the given signal proton analyzed (usually the largest and/or most well isolated signal is chosen). Correction factors of 1.55, 1.03 and 1.13, respectively, for tryptophan, *N*-acetylglucosamine and 3NPG were used to account for different relaxation properties. These factors have been obtained by dividing the integral ratio of the pyrazine peak over the ligand peak at d1 = 8 s by the same integral ratio at d1 = 3 s, from spectra obtained on homogeneous ligand samples. Uncertainties quoted in Table 1 are derived from the fitting error. The centre of the CSI image was determined as 20 mm from the absolute base of the NMR tube using biphasic samples as described in our previous work.[3] The top of the CSI analysis window (excluding the first slice) is given by 20 mm + 7 x 1.625 mm = 31.4 mm, which is where the boundary must be placed (corresponding to 400 uL of solution), as in Figure 2a of the main text. All spectra were referenced to pyrazine at 8.577 ppm.

ηSTDs, defined as in Equation S1 below, are manually calculated by shape matching, for each of the relevant protons for the analysis, at each concentration and each saturation time. More details on this procedure are reported in the following Section, Section S2.

S2. Mathematical derivation of STD-AF_0 and of K_D from the STD-AF_0 binding isotherm

The STD amplification factor (STD-AF), given by the intensity of an STD signal (ηSTD) corrected by the excess of ligand (expressed as the ratio of total ligand over total protein concentrations: $[L]_T/[P]_T$), gives indirect information about the concentration of the protein–ligand complex in solution. Therefore, STD NMR titration experiments can be used to get binding isotherms and derive ligand–receptor binding affinities, e.g., the K_D of the complexes as shown in Equation S3. In the binding isotherm approach, expressed in Equation S3 in relation to STD-AF([L]), we are under the assumption that the ligand excess is always large enough that we can approximate [L] to $[L]_T$. For low $[L]_T/[P]_T$ ratios, please see the mathematical derivation of the full law of mass action approach, reported in Section S5.

Equation S1 $$\eta_{STD} = \frac{I_0 - I_{Sat}}{I_0}$$

Equation S2 $$STD - AF = \frac{[L]_T}{[P]_T}\eta_{STD} \propto [PL]$$

Equation S3 $$STD - AF([L]) = \frac{\alpha_{STD-AF}[L]}{[L]+K_D}$$

As the effect of rebinding at longer saturation times can be remarkable and translates into potentially highly over-estimated K_Ds, it is necessary to determine binding isotherms and extract K_Ds from STD amplification factors at initial growth rates ($STD - AF_0$), rather than from single saturation times, to obtain the most accurate values. Equations S4 and S5, show how by derivative of the mono-exponential equation describing STD-AF as a function of t_{sat}, we can obtain $STD - AF_0$, and re-write Equation S3 as in Equation S6.

Equation S4 $$STD - AF(t) = STD_{max}(1 - e^{-k_{sat}t_{sat}})$$

Equation S5 $$STD - AF_0 = STD_{max}k_{sat}$$

Equation S6 $$STD - AF_0([L]) = \frac{\alpha_{STD\_AF0} * [L]}{[L]+K_D}$$

These steps have also been schematized in Figure 1 of the main text.

S3. Imaging STD NMR control experiments and comparison of STD NMR and Imaging STD NMR spectra

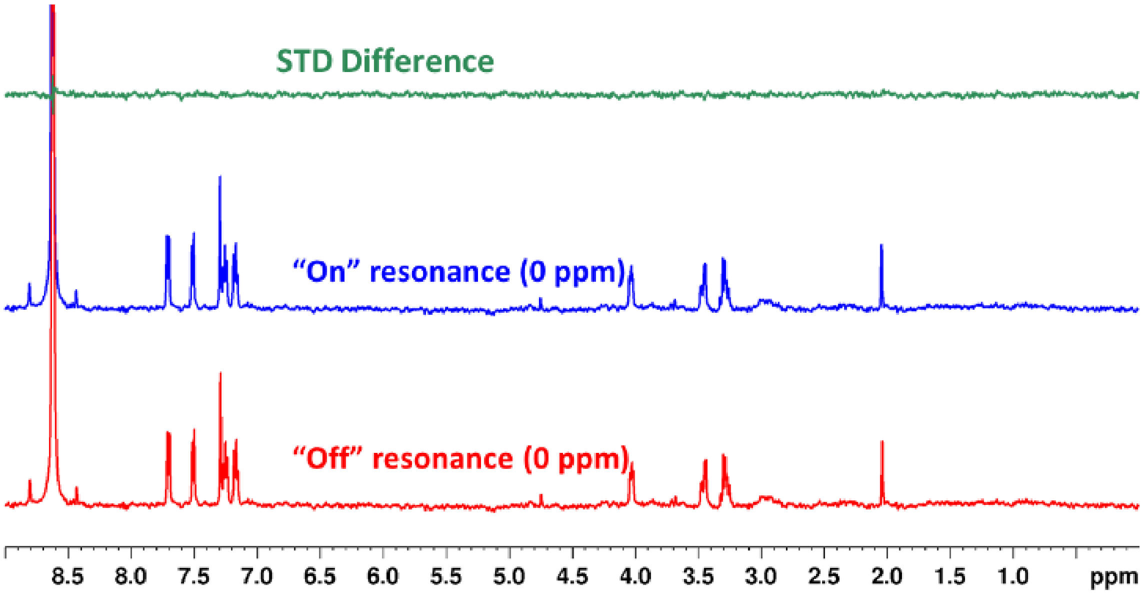

Figure S1. Spectra from an Imaging STD NMR control experiment, irradiating at 0 ppm both on resonance and off resonance. Same results were obtained for Imaging STD NMR experiments with on resonance and off resonance irradiation applied at both 40 ppm, and 0.87 ppm.

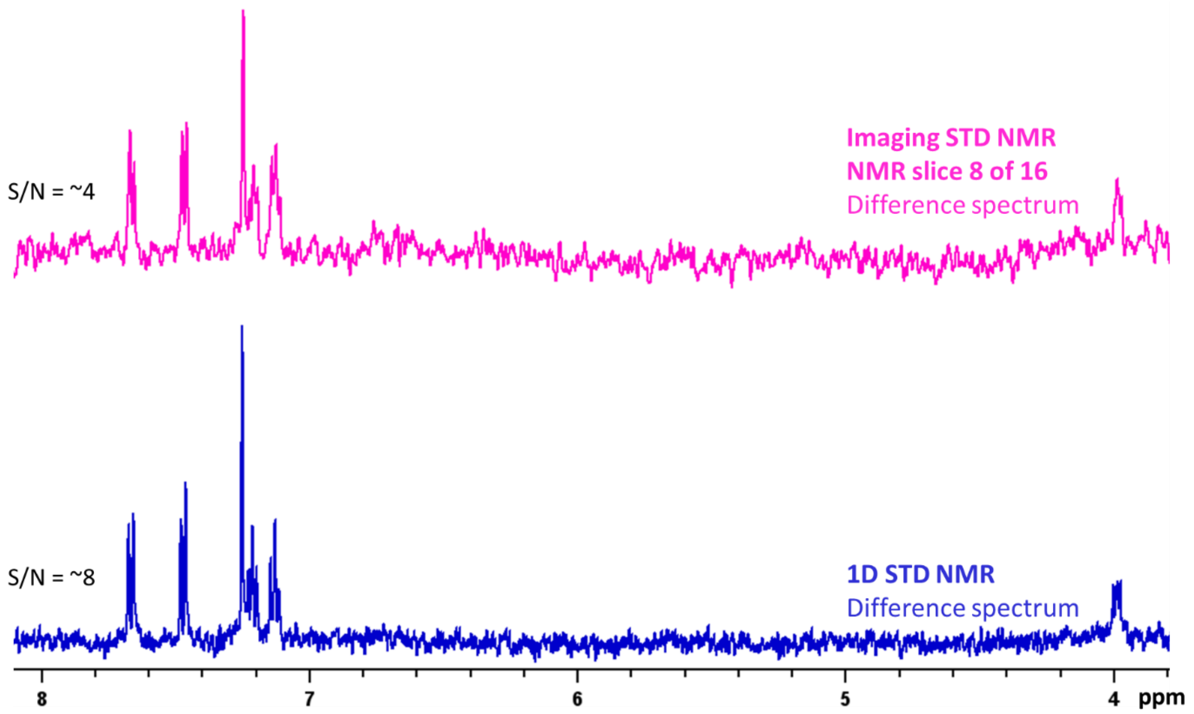

Figure S2. STD difference spectra of a homogeneous sample composed of 1.8 mM tryptophan and 50 μM BSA. Top: difference spectrum from slice 8 of 16 from an Imaging STD NMR experiment; bottom: 1D STD NMR difference spectrum (Bruker standard pulse sequence: stddiffesgp.3). Both experiments have been acquired in the same conditions (ns=8, d1 (relaxation delay)=3 s, d20 (saturation time) =1 s, T=298 K). The signal to noise ratio (S/N) is found to be double in the 1D STD NMR sample relative to the slice from Imaging STD NMR sample.

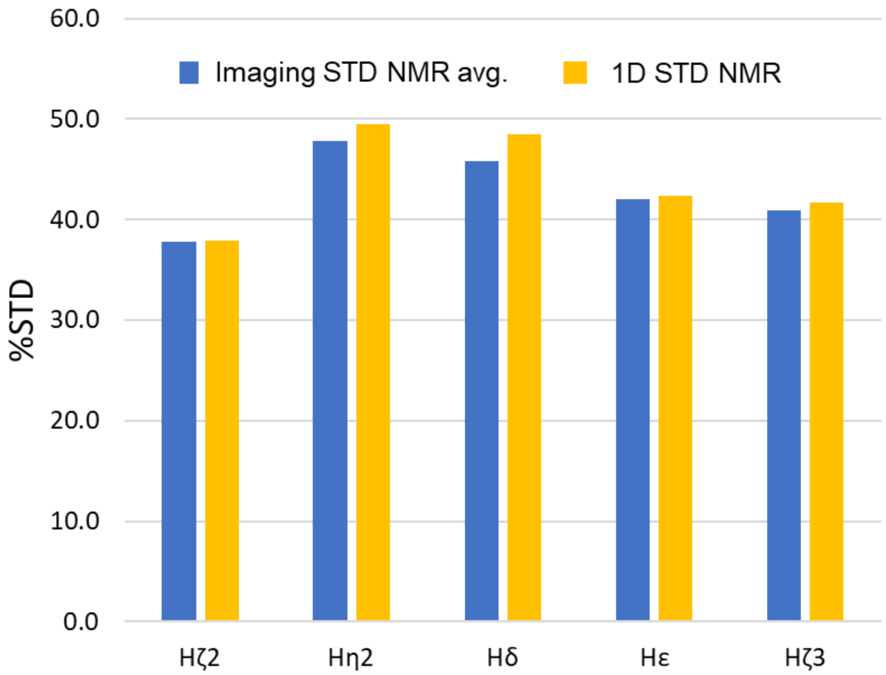

Figure S3. Histograms of the η_{STD} obtained from averaging the η_{STD} from each slice of Imaging STD NMR experiment (blue) and 1D STD NMR (yellow). Raw data in Table S1.

	η_{STD} for each proton of Tryptophan as bound to BSA				
**Slice**	**Hζ2**	**Hη2**	**Hδ**	**Hε**	**Hζ3**
**1**	38.43	48.57	46.71	47.17	41.55
**2**	38.81	48.17	45.81	41.55	41.12
**3**	38.06	48.57	44.93	40.85	41.49
**4**	37.32	48.57	45.47	42.36	41.95
**5**	37.06	48.1	45.2	40.28	41.89
**6**	37.69	47.26	45.82	41.15	41.37
**7**	38.06	48.57	46.26	40.19	40.49
**8**	38.81	47.17	45.37	40.35	39.96
**9**	37.69	47.82	45.03	40.01	41.3
**10**	38.06	47.17	47.4	41.75	41.55
**11**	37.6	47.19	47.65	41.87	39.51
**12**	37.5	47.27	45.46	43.29	38.11
**13**	37.32	47.28	45.37	43.63	40.6
**14**	36.2	47.1	45.17	43.97	41.37
**Avg**	**37.8**	**47.8**	**45.8**	**42.0**	**40.9**
**stdev**	0.7	0.6	0.8	1.9	1.0
**% err**	1.8	1.3	1.8	4.5	2.5
**STD NMR**	**37.96**	**49.53**	**48.53**	**42.37**	**41.64**

Table S1. Table of η_{STD} of the five aromatic protons of tryptophan from the same samples and experiments as in Figure S3, to assess the accuracy of the results obtained from an Imaging STD NMR experiment in comparison with a traditional STD NMR experiment in the same conditions. η_{STD} from each slice are reported and averaged, the standard deviation is calculated to be between 1% and 4%. The η_{STD} obtained from the 1D STD NMR experiments compare well from the average from the Imaging STD NMR experiment, deviation is within the experimental error.

S4. Build-up curves with tabulated data and binding isotherms tabulated data for the three complexes

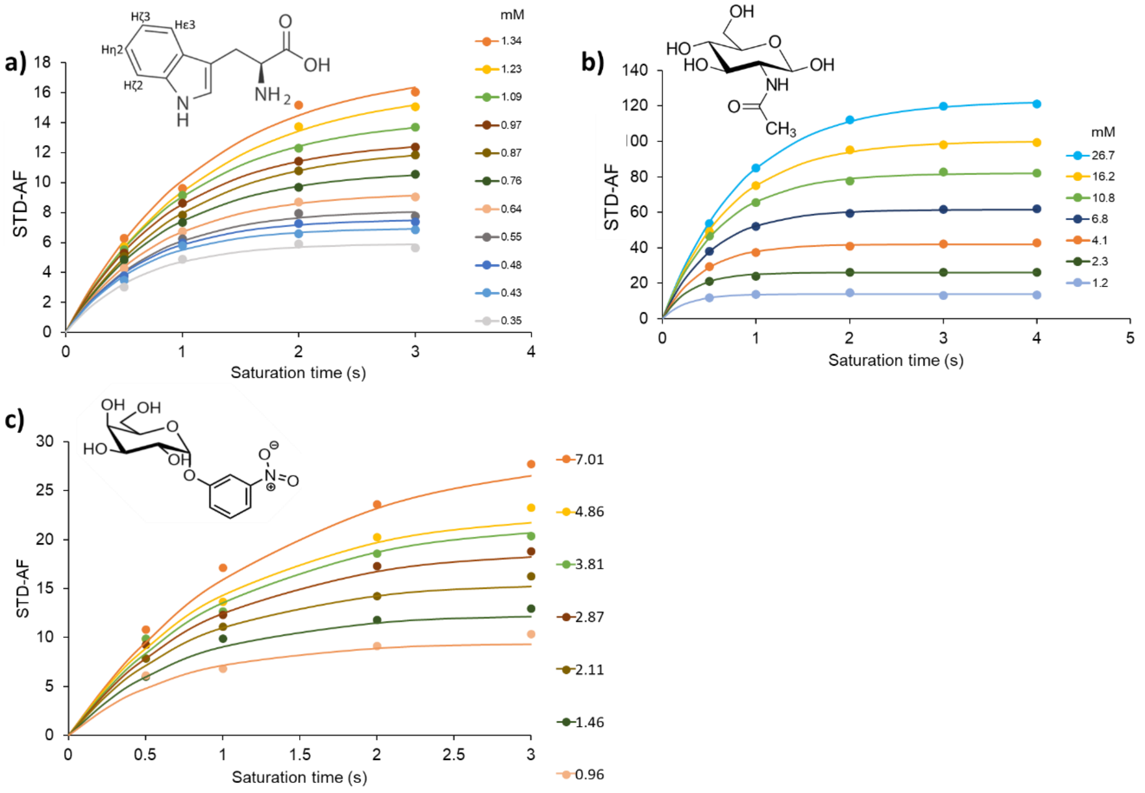

Figure S4. STD NMR build-up curves obtained at each depth of the tube, i.e. at increasing ligand concentration for W/BSA (**a**), GlcNAc/WGA (**b**) and 3NPG/CTB (**c**). For each curve, the fit is also shown. Tabulated data are reported in the tables below.

[W]/mM	0.5 s	1 s	2 s	3 s
**1.34**	6.27	9.61	15.13	16.03
**1.23**	5.72	9.15	13.73	15.04
**1.09**	5.50	9.17	12.27	13.69
**0.97**	5.29	8.63	11.41	12.37
**0.87**	5.07	7.82	10.78	11.82
**0.76**	4.84	7.33	9.68	10.55
**0.64**	4.26	6.73	8.68	9.03
**0.55**	3.75	6.26	7.95	7.74
**0.48**	3.80	5.99	7.27	7.36
**0.43**	3.50	5.81	6.59	6.84
**0.35**	3.02	4.89	5.92	5.63

Table S2. STD-AF (average for aromatic protons) build up curves for the W/BSA system.

[GlcNAc]/mM	0.5 s	1 s	2 s	3 s	4 s
**26.7**	53.79	84.92	112.27	119.85	121.10
**16.2**	49.15	75.21	95.12	97.98	99.23
**10.8**	46.57	65.40	77.76	82.89	82.00
**6.8**	37.95	52.11	59.42	61.53	61.89
**4.1**	29.32	37.30	40.90	42.09	42.67
**2.3**	21.20	24.02	26.10	26.05	26.03
**1.2**	11.66	13.5	14.52	13.12	13.51

Table S3. STD-AF build up curves for the methyl group for the GlcNAc/WGA system.

[3NPG]/mM	0.5 s	1 s	2 s	3 s	4 s
**7.0**	10.82	17.13	23.6	29.77	29.77
**4.9**	9.88	13.66	23.29	23.27	23.27
**3.8**	9.91	12.78	21.62	22.37	22.37
**2.8**	9.26	12.33	18.33	19.85	19.85
**2.1**	7.85	11.13	16.24	16.26	16.26
**1.5**	6.02	9.89	12.80	12.96	12.96
**0.95**	6.12	6.86	9.15	10.36	10.36

Table S4. STD-AF build up curves for the proton H2,3,5 of the sugar ring for the 3NPG/CTB system.

[W]/mM	0.5	1 s	2 s	3 s	STD-AF$_0$
**1.34**	0.72	0.63	0.43	0.34	0.87
**1.22**	0.66	0.60	0.39	0.32	0.80
**1.09**	0.63	0.60	0.35	0.29	0.84
**0.96**	0.61	0.57	0.32	0.26	0.92
**0.87**	0.58	0.51	0.31	0.25	0.75
**0.76**	0.56	0.48	0.27	0.22	0.74
**0.63**	0.49	0.44	0.25	0.19	0.69
**0.55**	0.43	0.41	0.22	0.16	0.65
**0.48**	0.44	0.39	0.20	0.16	0.67
**0.43**	0.40	0.38	0.19	0.15	0.64
**0.35**	0.34	0.32	0.17	0.12	0.55
**0.31**	0.36	0.29	0.14	0.11	0.60
**0.27**	0.35	0.26	0.12	0.10	0.61

Table S5. STD-AF values (average for aromatic protons) of the normalised binding isotherms, and initial growth rates (last column), for the W/BSA system (Figure 5a).

[GlcNAc]/mM	0.5 s	1 s	2 s	3 s	4 s	STD-AF$_0$
**26.7**	53.79	84.93	112.27	119.86	121.10	53.79
**16.2**	49.15	75.22	95.12	97.98	99.24	49.15
**10.8**	46.58	65.41	77.77	82.89	82.00	46.58
**6.8**	37.96	52.12	59.42	61.53	61.89	37.96
**4.1**	29.32	37.30	40.90	42.09	42.67	29.32
**2.3**	21.20	24.02	26.11	26.05	26.04	21.20
**1.2**	11.67	13.56	14.53	13.12	13.52	11.67

Table S6. STD-AF values of the normalised binding isotherms, and initial growth rates (last column), for the methyl group of GlcNAc/WGA system (Figure 5b).

[3NPG]/mM	0.5 s	1 s	2 s	3 s	STD-AF$_0$
**7.0**	10.83	17.13	23.66	29.77	22.91
**4.9**	9.88	13.66	23.30	23.28	22.39
**3.8**	9.91	12.70	21.63	22.37	21.05
**2.9**	9.27	12.33	18.34	19.85	20.21
**2.1**	7.86	11.14	16.25	16.27	19.03
**1.5**	6.03	9.89	12.81	12.97	16.28
**0.95**	6.13	6.86	9.15	10.37	13.41

Table S7. STD-AF values of the normalised binding isotherms, and initial growth rates (last column), for the methyl group of 3NPG/CTB system (Figure 5c).

S5. Mathematical derivation of more general fitting for K_D determination

The calculation of dissociation constants in this manuscript is based on the Langmuir isotherm. This relies on the approximation, which is almost always true in STD NMR studies, that the ligand excess is very high and so [L] in the Langmuir equation is approximately equal to the total concentration of ligand added. From the definition of K_D, we derive an expression that considers the total concentrations of protein and ligand, $[P]_T$ and $[L]_T$ respectively.

Equation S7 $$K_D = \frac{[P][L]}{[PL]}$$

Expressing [P] and [L] in terms of $[P]_T$, $[L]_T$ and the equilibrium concentration of protein ligand complex, [PL]:

Equation S8 $$[P] = [P]_T - [PL], \ [L] = [L]_T - [PL]$$

Equation S9 $$K_D = \frac{([P]_T - [PL])(L_T - [PL])}{[PL]} = \frac{[L]_T[P]_T - [PL][L]_T - [PL][P]_T + [PL]^2}{[PL]}$$

Rearranging as a quadratic equation for [PL]:

Equation S10 $$[PL]^2 - ([L]_T + [P]_T + K_D)[PL] - [L]_T[P]_T = 0$$

And using the quadratic solution:

Equation S11 $$[PL] = \frac{([L]_T + [P]_T + K_D) - \sqrt{([L]_T + [P]_T + K_D)^2 - 4[L]_T[P]_T}}{2}$$

Considering that:

Equation S12 $$STD - AF_0 \propto [PL]$$

We can include a proportionality parameter, β, and write STD-AF_0 as it follows, fitting for β and K_D:

Equation S13 $$STD - AF_0 = \beta \frac{([L]_T+[P]_T+K_D)-\sqrt{([L]_T+[P]_T+K_D)^2-4[L]_T[P]_T}}{2}$$

In Figure S5, we are showing the mathematical fits obtained for the three systems, and in Table S8 we report the K_Ds obtained through this approach, in comparison to the original Langmuir isotherm fitting, showing that the deviation is within the fitting error.

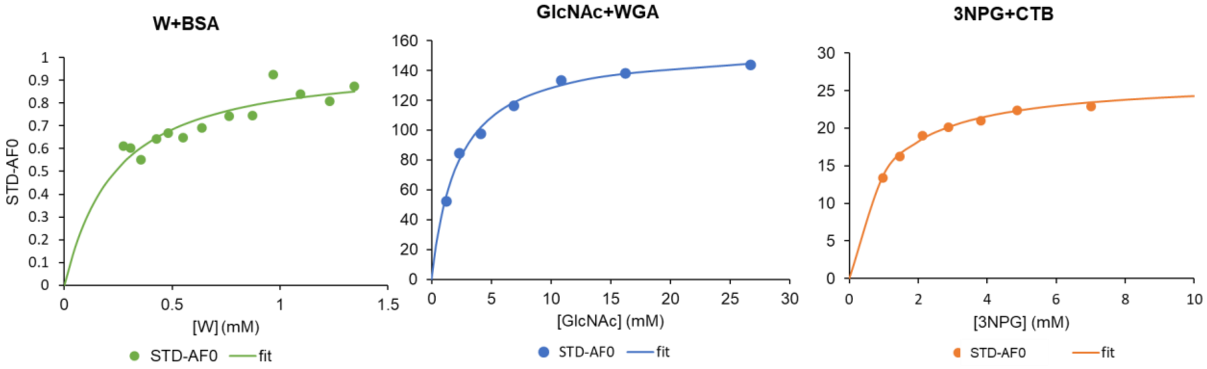

Figure S5. Mathematical fit obtained from the nonlinear least-squares fitting of the dissociation constant derived from the law of mass action (Equation S13), for the three systems in analysis.

K_Ds	3NPG+CTB	GlcNAc+WGA	W+BSA
Langmuir	0.23 mM	2.22 mM	0.93 mM
Mass action	0.20 mM	2.19 mM	0.90 mM

Table S8. Comparison of the K_Ds obtained for both systems using the Langmuir isotherm and the Law of mass action.

S6. Tables of K_Ds at single saturation times

	K_D/mM					
	W/BSA		GlcNAc/WGA		3NPG/CTB	
**STD-AF0**	0.23	±0.05	2.24	±0.18	0.93	±0.07
**0.5 s**	0.63	±0.08	4.76	±0.35	1.31	±0.27
**1 s**	0.73	±0.05	8.01	±0.37	1.77	±0.39
**2 s**	1.96	±0.26	12.04	±0.32	2.25	±0.34
**3 s**	2.51	±0.30	13.17	±0.66	3.30	±0.49

Table S9. Table of K_Ds fitted from initial slope (STD-AF0, first row) in comparison with K_Ds fitted from single saturation times. This table shows how, in extreme cases where build-up curves cannot be acquired due to low protein availability or small STD factors observed, a single saturation time experiment can still be used for obtaining the order of magnitude of the K_D, keeping in mind that it will provide an upper limit for the K_D.

S7. Table of comparison for instrument time for STD NMR titration and Imaging STD NMR

In Table 1 of the main text, we have compared the total instrument time required to run our Imaging STD NMR experiment for K_D determination with the instrument time required to run the build-up curves at each $[L]_T$:$[P]_T$ ratio in the manual STD NMR titration reported in [4]. These calculations were done taking into account the number of $[L]_T$:$[P]_T$ ratios assessed, number of saturation times, delays and number of scans (ns) of the manual titrations in [4], so to directly compare the K_Ds obtained by Imaging STD NMR with those previously obtained by Angulo *et al.*

We here want to compare the Imaging STD NMR titration time, with the correspondent time which would be required to run the same experiments (with same number of scans, delays, $[L]_T$:$[P]_T$) manually. In Table S8, for each system, we indicate number of scans (ns) run for each saturation time experiments, the respective experimental times ("expt."), required to obtain a build-up curve and we modify it by the number of $[L]_T$:$[P]_T$ assessed, to give the total instrument time for each titration. For manual titrations, to the instrument time, we need to add the time required to recover the sample, carry out the ligand addition, allow 30-40 minutes for ligand dissolution and complex equilibration, re-injection of the sample, optimisation of the spectrometer, which we call "waiting time" in the table, multiplied again for number of $[L]_T$:$[P]_T$ points. We call this the "Manual titration time", which we compare to "Imaging STD titration time".

T_{sat}<br>W	0.5s	1s	2s	3s	4s	No. $[L]_T$:$[P]_T$ point	Total instrument time (h)	Waiting time (h)	Manual titration time (h)	Imaging STD titration time (h)
**ns**	16	16	8	8		13				
**Expt (min)**	5	5	2.5	2.5			3.25	1	19.5	2.5
**GlcNAc**										
**ns**	16	8	8	8	8	7				
**Expt (min)**	6	3	3	3	3		1.4	1	10.5	2
**3NPG**										
**ns**	32	16	8	8		7				
**Expt (min)**	10	5	2.5	2.5			2.4	1	11.7	2.75

Table S10. Comparison of the instrument and total titration time required for both manual STD NMR and Imaging STD NMR titration for K_D determination.

Remarkably, for manual STD NMR titration an operator is required to constantly attend the sample; instead, in our approach, the sample can simply be prepared the day before and let to develop over night to be analysed in a single go and unattended, the following morning. Also, in Section S13, we are providing scripts for fully automated data acquisition and processing, to further speed up the process.

Finally, and most importantly, we believe that the constant manipulation of the sample required with the manual titration is among its most significant drawbacks, and that in fact the cleanliness of the experiment, deriving from the fact that i) the sample is just prepared and sealed, and ii) that the concentrations are internally referenced, constitutes the main advantage of Imaging STD NMR.

Another advantage of Imaging STD NMR over the manual STD NMR titration is the ligand consumption. In Table S11, we report, fixing the concentration of each ligand stock, the volume required to achieve the maximum concentration analysed for each of the three system. Depending on the systems, the saving varies between 50% and 85%. Whereas this may not always be a problem in industrial settings where often leads are synthesized in large amounts, the amount of ligand required can definitely be a limitation when it comes to physiological substrates.

	$[L]_{STOCK}$ (mM)	$[L]_{MAX}$ (mM)	Volume Manual STD NMR (μL)	Volume Imaging STD NMR (μL)	Ligand saving
W	37.5	1.34	18	9	50%
GlcNAc	50	26.7	267	50	70%
3NPG	18	7	194	32	85%

Table S11. Calculations of ligand consumption for manual STD NMR titration and Imaging STD NMR titration, where $[L]_{stock}$ is the concentration of the ligand stock used for the analysis, and $[L]_{max}$ is the maximum concentration that we obtained in at the closest point to the boundary analyses. The volume columns refer to the amount of ligand stock used in the gradient formation in the Imaging STD NMR titration, and the volume which would have been necessary to reach the same $[L]_{max}$ in the manual titration.

S8. Binding epitope mappings at increasing concentrations for W/BSA and GlcNAc/WGA

[W]/mM	Hζ2	Hη2	Hδ	Hε	Hζ3
**0.64**	99	73	77	94	100
**0.76**	89	69	69	77	100
**1.23**	99	92	85	89	100
**1.34**	99	86	75	91	100
**2.81**	80	77	59	78	100
**3.84**	83	100	81	99	91
**4.33**	82	85	82	84	100
**5.34**	87	88	87	94	100
**6.26**	81	84	78	100	93
**7.35**	75	100	79	95	95
**8.62**	77	100	76	87	100

Table S12. Binding epitope mappings from STD NMR for 5 tryptophan protons at increasing ligand concentration. Each binding epitope is obtained by normalisation against the highest signal – these change from one concentration to the other due to loss of the binding epitope pattern above 2 mM.

[GlcNAc]/mM	CH3	H2_β	H4_β	H3_α	H6_β
**4.8**	100	7.73	8.71	8.59	8.59
**7.2**	100	10.9	6.40	8.45	8.41
**9.9**	100	11.9	11.6	9.96	9.93
**12.8**	100	10.3	10.7	8.75	8.75
**16.1**	100	9.57	9.31	10.1	10.1
**19.4**	100	10.6	12.5	9.70	9.70
**22.7**	100	9.69	10.3	10.8	10.8
**26.1**	100	9.36	12.0	9.15	9.15
**33.4**	100	8.40	13.2	10.9	9.07

Table S13. Binding epitope mappings from STD NMR for 5 GlcNAc protons at increasing ligand concentration. Each binding epitope is obtained by normalisation against the highest signal, in this case consistently the methyl group.

[3NPG]/mM	Ha	Hb	Hc,d	H1	H4	H2,3,5
**7.62**	96	65	34	62	74	100
**5.05**	96	58	31	57	72	100
**3.79**	93	49	25	46	79	100
**2.63**	72	49	19	45	80	100
**1.70**	72	50	26	51	70	100
**1.08**	68	47	23	51	69	100
**0.78**	61	53	21	53	78	100

Table S14. Binding epitope mappings from STD NMR for 6 3NPG protons at increasing ligand concentration. Each binding epitope is obtained by normalisation against the highest signal, in this case consistently the H2,3,5 of the sugar ring.

S9. Pulse sequence for STD CSI experiment (Bruker)

The on- and off-resonance experiments are stored in two different experiment folders (so for each experiment a “mulexpno” experiment folder should be created and linked to. The difference spectra are obtained by the difference of the “on-resonance – off-resonance experiment” at each slice. TopSpin macros have been written to facilitate the process of automatized processing, referencing and generating the different spectra (Section S13).

```
;Modified from: "Probing spatial distribution of alignment by deuterium NMR imaging"
;Chemistry - A European Journal, Volume 19, Issue 22, 27 May 2013, Pages 7013-7019
;2D sequence for z-imaging preserving chemical shift. DOI: 10.1002/chem.201300254
;using a phase encoding gradient. Original sequence written by Christian Merle, Martin Koos
;Modified to be on 1H with perfect echo excitation sculpting for water suppression
;Presaturation of protein using train of shaped pulses
;Water suppression component is taken from:
;zgesgppe
;avance-version (13/08/01)
;1D sequence
;water suppression using excitation sculpting with gradients
;   using perfect echo
;(R.W. Adams, C.M. Holroyd, J.A. Aguilar, M. Nilsson & G.A. Morris,
;   Chem. Commun. 49, 358-360 (2013))
;T.-L. Hwang & A.J. Shaka, J. Magn. Reson.,
;   Series A 112 275-279 (1995)
;
;Serena Monaco and Matthew Wallace, 9/2022 (University of East Anglia, matthew.wallace@uea.ac.uk)
;This pulse program is not fully tested and comes without warranty.
;Check the your parameters and the sequence carefully before use.
;Presaturation frequency is set by cnst20 (on-resonance) and cnst21 (off resonance). These are in terms of frequency (Hz) from o1.
;o1 is normally set to frequency of water for water suppression. Upfield is -ve frequency
;Set 1 SW to Z-range in mm (see cnst0) to get 1 Hz/mm scale in indirect dimension when plotted
;Make cnst0 bigger than actual sample size to avoid folding artefacts.
;Keep gpz6 at 100% and adjust cnst3 to get p30 to an acceptable length according to your instrument (ca. 100-300 us)
;Set MULEXPNO[1] to experiment number to contain the off resonance dataset
;On-resonance is stored in the dataset executed
; 1H-Version
;$CLASS=HighRes
;$DIM=2D
;$TYPE=
;$SUBTYPE=
;$COMMENT=
prosol relations=<triple>
#include <Avance.incl>
#include <Grad.incl>
#include <Delay.incl>
"cnst2= 0.8914027" ; integralfactor of gradient shape
"cnst4= 267.52220" ; * 10^6 /Ts = gamma1H
"p30=(td1/cnst0) * (1/(cnst1*cnst2*cnst3)) * (1/cnst4) *(2*3.14159265/1000)* 0.5 s"
"l1=td1-1"
lgrad r1d = l1
"acqt0=0"
;1 cm equivalent to 10 Hz
"DELTA3=p30+d16"
"d12=20u"
"l5=d20/p29"
"d31=p29*l5"
"DELTA1=p12+p16+d16+p2/2+de/2+p1/PI+12u"
"DELTA2=d1-d31"
"TAU=de+p1*2/PI"
"p2=p1*2"
baseopt_echo

1 ze
30m
30m
30m
2 30m
3 50u BLKGRAD
DELTA2
;Shift away from SFO1
d12 fq=cnst20:f1
10 (p29:sp2 ph29):f1
4u
;Shift back again
lo to 10 times l5
d12 fq=0:f1
50u UNBLKGRAD
p19:gp4
d16
d12 pl1:f1
p1 ph1
  p16:gp3
  d16
  DELTA1
  (p2 ph7)
  DELTA1
  p16:gp3
  d16

  (p1 ph6)

  p16:gp1
  d16
  (p12:sp1 ph2:r):f1
```

```
  4u
  4u pl1:f1

  p2 ph3

  4u
  p16:gp1
  d16
  TAU
  p16:gp2
  d16
DELTA3
  (p12:sp1 ph4:r):f1
  4u
  4u pl1:f1

  p2 ph5

  4u
  p16:gp2
  d16
;end of block
p30:gp6*r1d*cnst3
d16
go=2 ph31
30m wr #0 if #0 zd
goto 22
4 30m
22 50u BLKGRAD
DELTA2
;Shift away from SFO1
d12 fq=cnst21:f1
12 (p29:sp2 ph29):f1
4u
;Shift back again
lo to 12 times l5
d12 fq=0:f1
50u UNBLKGRAD
p19:gp4
d16
d12 pl1:f1
p1 ph1
  p16:gp3
  d16
  DELTA1
  (p2 ph7)
  DELTA1
  p16:gp3
  d16

  (p1 ph6)

  p16:gp1
  d16
  (p12:sp1 ph2:r):f1
  4u
  4u pl1:f1

  p2 ph3

  4u
  p16:gp1
  d16
  TAU
  p16:gp2
  d16
DELTA3
  (p12:sp1 ph4:r):f1
  4u
  4u pl1:f1

  p2 ph5

  4u
  p16:gp2
  d16
;end of block
p30:gp6*r1d*cnst3
d16
go=4 ph31
;only increment gradient after doing n scans at one gradinet strength,
;at both presat frequencies
30m wr #1 if #1 zd igrad r1d
lo to 3 times l1
goto 5
; run last increment:
8 30m
5 50u BLKGRAD
DELTA2
;Shift away from SFO1
d12 fq=cnst20:f1
11 (p29:sp2 ph29):f1
4u
lo to 11 times l5
;Shift back again
d12 fq=0:f1
50u UNBLKGRAD
p19:gp4
d16
d12 pl1:f1
p1 ph1
```

```
  p16:gp3
  d16
  DELTA1
  (p2 ph7)
  DELTA1
  p16:gp3
  d16

  (p1 ph6)

  p16:gp1
  d16
  (p12:sp1 ph2:r):f1
  4u
  4u pl1:f1

  p2 ph3

  4u
  p16:gp1
  d16
  TAU
  p16:gp2
  d16
DELTA3
  (p12:sp1 ph4:r):f1
  4u
  4u pl1:f1

  p2 ph5

  4u
  p16:gp2
  d16
;end of block
p30:gp6*r1d*cnst3
d16
go=8 ph31
30m wr #0 if #0 zd
;spoil gradient from previous
;Shift away from SFO1
goto 23
9 30m
23 50u BLKGRAD
DELTA2
d12 fq=cnst21:f1
13 (p29:sp2 ph29):f1
4u
lo to 13 times l5
;Shift back again
d12 fq=0:f1
50u UNBLKGRAD
p19:gp4
d16
d12 pl1:f1
p1 ph1
  p16:gp3
  d16
  DELTA1
  (p2 ph7)
  DELTA1
  p16:gp3
  d16

  (p1 ph6)

  p16:gp1
  d16
  (p12:sp1 ph2:r):f1
  4u
  4u pl1:f1

  p2 ph3

  4u
  p16:gp1
  d16
  TAU
  p16:gp2
  d16
DELTA3
  (p12:sp1 ph4:r):f1
  4u
  4u pl1:f1

  p2 ph5

  4u
  p16:gp2
  d16
;end of block
p30:gp6*r1d*cnst3
d16
go=9 ph31
30m wr #1 if #1 zd
30m BLKGRAD
exit
ph1=0
ph2=0 1
ph3=2 3
ph4=0 0 1 1
```

```
ph5=2 2 3 3
ph6=1
ph7=0
ph29=0
ph31=0 2 2 0


;cnst0 : z-Range in cm
;cnst1 : GCC (G/mm) from Gradpar
;cnst3 : set to get P30 of acceptable length
;cnst20: Signed distance Hz from water frequency (upfield -ve) to saturate
;cnst21: Off res presat Hz
;pl1 : f1 channel - power level for pulse (default)
;p1 : f1 channel - 90 degree high power pulse
;p12: sp1 shaped pulse for water suppression
;p16 : watergate gradient pulse
;p19 : Spoil gradient pulse (1000 us)
;p27: f1 channel -  90 degree pulse at pl18
;p29: sp2 shaped pulse for presat [50m]
;gpz4: spoil gradient [50%]
;gpz6: 100% phase encoding gradient
;d12: delay for power switching                    [30 usec]
;d16: standard eddy delay (200u)
;ns: 8 * n, total number of scans: NS * TD0
;ds: 4
;d20: saturation time
;d31: saturation time as executed
;d1 : relaxation delay; 1-5 * T1
;l5: loop for saturation: p29 * l5 = saturation time
;td1: number of experiments
;spw1 : power of water suppression pulse
;spw2 : power of saturation pulse
;FnMODE: QF

;for z-only gradients:
;gpz1: 31%
;gpz2: 11%
;gpz3: 5%

;use gradient files:
;gpnam1: SMSQ10.100
;gpnam2: SMSQ10.100
;gpnam3: SMSQ10.100
;gpnam4: SMSQ10.100
;gpnam6: SMSQ10.32

;$Id: phaseenc,v 1.1 2011/08/10 15:12:45 ber Exp $"
```

S10. Excel spreadsheet for amount of mass and concentration for gradient formation of small molecules

It is here reported an example of the spreadsheet used to calculate exact mass and therefore concentration to add at the top of the tube to obtain ligand's gradients across the Z axes of the tube. The equation used is the following:

$$m = \frac{C(z)\,\pi r^2 MW\sqrt{\pi D t}}{e^{-z^2/4Dt}}$$

This is an extract of the Excel sheet, which is also included a separate Excel file for user consultation. This calculation is set for tryptophan, where a concentration of 3 mM is required at 4 mm (z) from the top of the tube 18 h (64800 s) after sample preparation. The concentration of the tryptophan stock in used is 37.5 mM, so the outcome is the volume to add at the top of the tube. Alternatively, the volume can be fixed and the concentration of the stock calculated.

D	5E-10	m^2/s				
m	0.00065835	g	1 mg			
r	2.1	mm	5 mm			
D	0.0005	mm^2/s				
MW	204.43	g/mol				
t	64800	s	18	h		
π	3.14159					
z	4	mm				
C mM	3	C (mol/mm^3)		0.000000003		
	denominator	nominator				
	0.88385983	8.57238E-05				
m	0.0000970	g	0.09698	mg	mol	4.74431E-07

Setting C

V	12.7	uL
V	1.2651E-05	L
C	37.5	mM
C	0.0375	M

In conclusion of this section, it is worth mentioning that this approach works very well for calculating the concentration at the top of the tube (maximum concentration) but it is not as efficient at calculating the concentration at the bottom of the tube (minimum concentration). This is to be ascribed to an NMR tube not being an infinite cylinder: therefore, at the bottom of the tube we have higher concentration than what theoretically expected, due to the "re-bouncing" effect, once the ligand molecules reach the edge of tube. However, as we have demonstrated, setting the maximum concentration of the window is sufficient to obtain gradients suitable to obtain optimal binding isotherms from Imaging STD NMR.

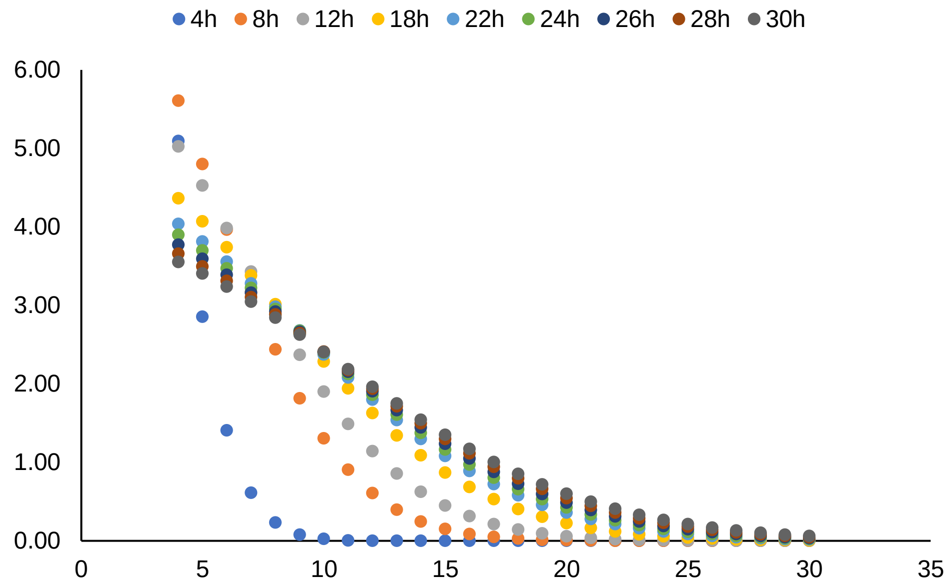

Figure S6. Simulated diffusion profile of tryptophan with time, showing that the gradient is essentially stable from 22 to 26 hours.

S11. Effect of DMSO in the stock of diffusion profile

In this section, we explore the scenario, common to organic ligands, of poor water solubility. Indeed, in many cases, organic ligands require some DMSO content for full solubilisation. A DMSO-containing ligand stock would have higher density than the buffer and therefore could not be placed on top of it. In Figure S7, we demonstrate that the diffusion profile obtained by spinning a small amount of DMSO-containing stock at the bottom of the tube and carefully layering on top a solution containing buffer and reference, does look exactly like the diffusion profile of the gradient obtained by placing a D_2O stock on top of the buffer.

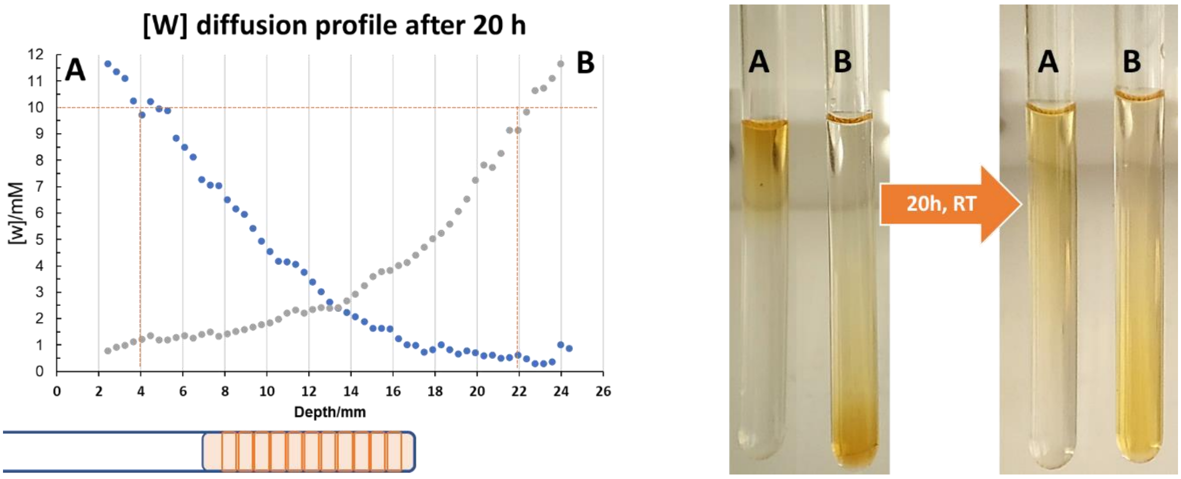

Figure S7. Gradient profiles of samples A and B, obtained by downward (from D_2O) and upward (from 50:50 D_2O:DMSO-d6) diffusion, respectively. In the left panel we have diffusion profile of both samples, while in the right panel we have pictures of the two samples at time 0 and time 20h. 0.5 mM methyl orange has been included in the stock solutions, to follow diffusion visually.

The samples in Figure S7 have been prepared following the calculations from the spreadsheet and equation in the previous Section S10, aiming at a concentration of 10 mM at 4 mM from the boundary (either bottom or top), after 20 h. Therefore, for sample A, we placed 45 µL of tryptophan 37.5 mM in D_2O on top of 400 µL of buffer and reference solution, while for sample B, we placed 45 µL of tryptophan 37.5 mM in 50:50 D_2O:DMSO-d6 at the bottom of the tube, layering 400 µL of buffer and reference solution on top, and let develop for 20 hours. We can see both numerically and visually that the diffusion profile is not affected by the direction of diffusion and the presence of DMSO in sample B.

It is important to highlight at the same time, that the use of a D_2O:DMSO-d6 50:50 solution for a ligand is an extreme condition which would not be suitable for most protein. In fact, at high DMSO-d6 concentration the proteins would precipitate due to denaturation in the proximity of the boundary invalidating the experiment. This example is only used as a proof of concept to show that DMSO presence in the stock solution, in case of solubility problems, is tolerated and it does not pose limitations to the applicability of our technique.

S12. Effect of protein concentration on sensitivity and simulation of protein diffusion

Figure S8 reports the STD NMR difference spectra acquired on homogeneous samples at decreasing protein concentration of BSA (from 50 µM BSA at the bottom, to 6.25 µM BSA M at the top) in the presence of a constant 1 mM tryptophan concentration. In each section of the figure, the blue track corresponds to the 1D STD NMR difference spectrum, while the pink track corresponds to the slice 8 of 16 of our Imaging STD NMR experiment, similarly to what is reported in Figure S2. The S/N ratio of the slice from Imaging STD NMR is found to be respectively 30% in the 6.25 µM sample, 40% in the 12.5 µM sample, 50% in the 25 µM sample and 50% in the 50 µM, relative to the 1D STD NMR experiment.

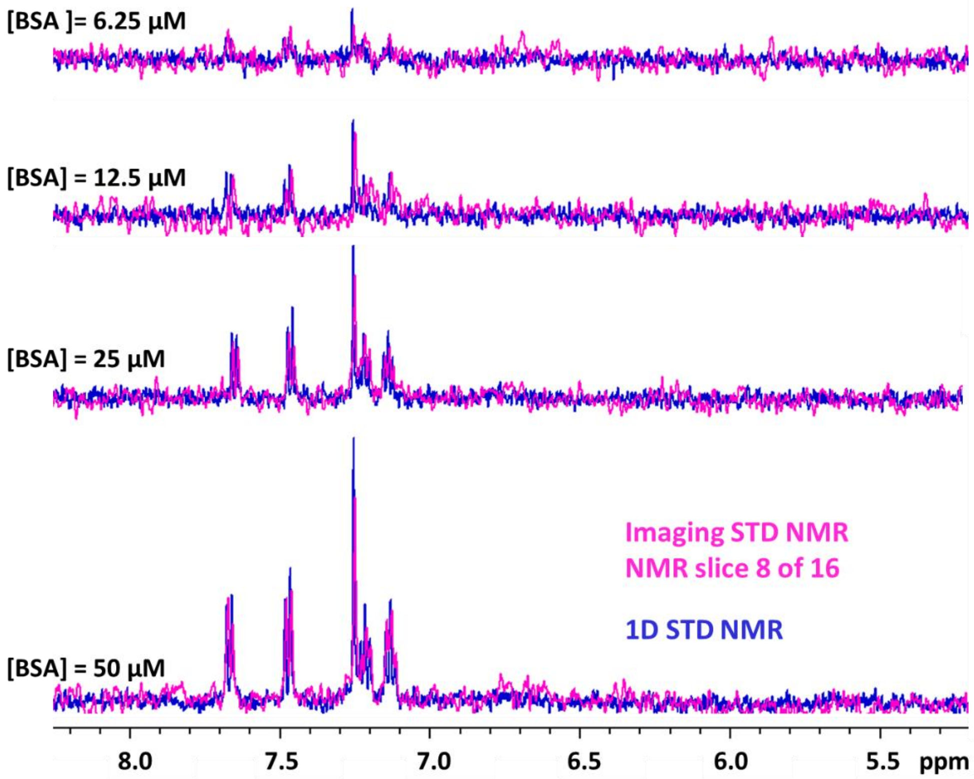

Figure S8. STD NMR difference spectra from 1D STD NMR experiments (in blue) and Imaging STD NMR experiments (in pink), on homogeneous 1 mM tryptophan samples, at decreasing

protein concentrations, from 50 μM BSA at the bottom to 6.25 μM BSA at the top. For all the experiments, saturation time = 1 s, d1 = 3 s, and ns = 8.

The results show that the sensitivity of the experiments is enough to decrease the protein concentration (down to at least 4- to 5-fold dilution, if the STD NMR signal is still detectable), bearing in mind that it has already been shown that, using the initial slope approach, the effect of protein concentrations on the K_D calculations is negligible.[4]

Finally, we analyzed the possibility of protein diffusion towards the top of the tube, when the small volume of ligand stock is carefully layered on the top of the solution containing the protein. To that aim, we ran some simulations to assess the extent of diffusion to be expected at increasing time points, as shown in Figure S9.

If the column of ligand solution placed on top of the protein solution was infinitely long, the total concentration of protein at distance above the boundary of the two solutions would be given by:[5]

$$[\mathrm{P}]_\mathrm{T} = 0.5\mathrm{C}_0\mathrm{erfc}\left(\frac{\mathrm{z}}{2\sqrt{\mathrm{Dt}}}\right)$$

where C_0 is the initial concentration of protein in the solution (50 μM for BSA), z is the distance above the boundary of the two solutions (boundary is 31.4 mm from the base of the NMR tube) and D is the diffusion coefficient, taken as 5 x 10^{-11} m^2s^{-1} for BSA.[6] Calculating the concentration of BSA, the upwards diffusion of the protein only affects the top three useable slices in the dataset which will be at the higher ligand concentration. In practice, the ligand solution is not infinitely long (< 4 mm for 50 μL of ligand solution, tube radius of 2.1 mm) so the loss of protein by diffusion will considerably be less. From Figure 5, the three points at higher ligand concentration, corresponding to those closest to the boundary, lie on the fitted line in agreement with the other points. We thus conclude that upwards diffusion of protein into the ligand solution does not significantly affect our fitted values of K_D. However, we recommend that the ligand solution is no more than 50 μL to minimize the upwards loss of protein by diffusion, or else the ligand solution is prepared with the same concentration of protein as the lower solution.

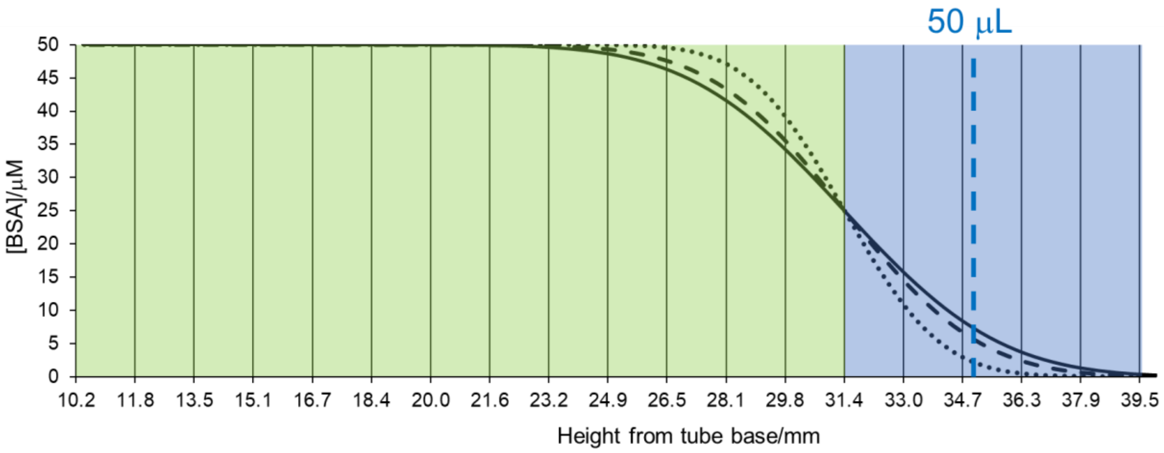

Figure S9. Plot of BSA concentration versus height from base of NMR tube 12 (dotted), 24 (dashed) and 32 hours (solid line) since ligand solution (blue) was placed on top of 50 μM BSA (green). Boundary of protein and ligand solutions initially at 31.4 mm. Vertical bars separate are 1.63 mm apart like the slices in the CSI dataset. Height of 50 μL of ligand solution is indicated, protein above this position would be ‘reflected’ back onto the lower slices.

S13. Macros for automated Imaging STD NMR data processing (Bruker)

S13.1 Macro for automatic phasing, baseline correction and referencing.

This Bruker automation script is a macro allowing automatic phasing, baseline correction, referencing of each slice of the spectrum and splitting each slice in a consecutive process number (procno). Several sequential CSI experiments can be processed with one run of this script:

```
/*To process multiple sequential 2D CSI datasets in sequential experiment numbers*/
/*If CSI datasets obtained at several saturation times*/
/*To produce phase corrected chemical shift image from gradient encoded data*/
/*Set 1st order phase correction for f1 dimension to 180*Td1 (2880 when 16 points in dataset), 0th order to 0*/
/*PH_mod should be set to PK in both dimensions*/
/*XFB to produce image*/
/*With the first 2D dataset selected, Run this Au*/
/*AU extracts each row in turn to a procno and automatically phase, baseline corrects*/
/*and references to pyrazine (or any another resonance) if requested*/
/*then advances to next sequential experiment number and does the same*/
/*Reversal of F1 axis may be necessary to get rows the write way up, depending on NMR probe*/
/*This AU is not fully tested and comes without warranty.*/
/*The script works on Bruker Topspin 3.6.2 but has not been tested on other versions*/
/*Use kill command if all goes wrong*/
/*Serena Monaco and Matthew Wallace, 9/2022*/
/*University of East Anglia, matthew.wallace@uea.ac.uk*/
char disk1[32], user1[32], location[128], phtyp[8],extractans[8],refans[8];
float abf1=8;
float abf2=6;
float lb=3;
float min=0;
double f2pref=8;
double f1pref=9;
double ref=8.577;
double o1,bf1;
double ppsens=0.9;
double pc=0.1;
double peakFreqHz,sfn, peakFreqPPM, peakIntensity, maxpsh;
double maxpsp, maxips, sf,sfo1,so1p,mintpp,minpsp,peakppmneg,cent,ppmdif,maxpspneg;
int i, numPeaks;
int phpno=1;
int w=1;
int np=64;
int pno=5;
int ne=1;
int m=-1;
int extractno=2;
int extrctv=1;
GETCURDATA
int steno=expno;
strcpy(location,disk);
strcpy(phtyp,"k");
strcpy(extractans,"y");
strcpy(refans,"y");
GETSTRING("Enter location of dataset",location)
phpno=procno;
GETINT("Enter first experiment number to process",steno)
GETINT("Enter number of 2D experiments to process",ne)
GETINT("Enter procno containing 2D data :",phpno)
pno=phpno+998;
GETINT("Enter procno to write rows to phase and baseline correct (empty):",pno)
GETSTRING("Extract phased rows?",extractans)
if(strcmp(extractans,"y")==0)
{
GETINT("Enter procno to contain 1st row of phased data:",extractno)
extrctv=extractno;
GETSTRING("Reference extracted rows?",refans)
if(strcmp(refans,"y")==0)
{
GETDOUBLE("Enter left limit for reference/ppm:",f1pref)
GETDOUBLE("Enter right limit for reference/ppm:",f2pref)
GETDOUBLE("Enter chemical shift of reference compound/ppm:",ref)
}
}
REXPNO(steno)
RPROCNO(phpno)
SETCURDATA
FETCHPAR1("SI",&np)
FETCHPAR("lb",&lb)
GETINT("Enter number of points in image (autodetects) :",np)
GETSTRING("APKS (s) or APK (k) or apkf (f) auto phase correction?",phtyp)
if(strcmp(phtyp,"f")==0)
{
GETFLOAT("Enter right limit for apkf and absf:",abf2)
GETFLOAT("Enter left limit for apkf and absf:",abf1)
}
w=1;
GETFLOAT("Enter line broadening factor (Hz):",lb)
TIMES(ne)
{
```

```
REXPNO(steno)
RPROCNO(phpno)
SETCURDATA
STOREPAR("lb",lb)
XFB
TIMES2(np)
{
RPROCNO(phpno)
SETCURDATA
RSR(w,pno)
RPROCNO(pno)
SETCURDATA
if(strcmp(phtyp,"s")==0)
{
APKS
ABS
}
if(strcmp(phtyp,"k")==0)
{
APK
ABS
}
if(strcmp(phtyp,"f")==0)
{
STOREPAR("absf1",abf1)
STOREPAR("absf2",abf2)
APKF
ABSF
}
WSR(w,phpno,steno,name,user,location)
if(strcmp(extractans,"y")==0)
{
if(strcmp(refans,"y")==0)
{
STOREPAR("f2p",f2pref)
STOREPAR("f1p",f1pref)
STOREPAR("mi",min)
STOREPAR("pc",pc)
/*Use global scaling to avoid errors about scal reg file*/
STOREPAR("pscal",1)
PP
 numPeaks = readPeakList(PROCPATH(0));
maxips=0.0;
    maxpsh=0.0;
    for (i=0; i<numPeaks; i++)
    {
                    peakIntensity = getPeakIntensity(i);
                    peakFreqHz = getPeakFreqHz(i);
                    peakFreqPPM = getPeakFreqPPM(i);
                    if (peakIntensity > maxips)
                    {
                        maxips = peakIntensity;
                        maxpsh = peakFreqHz;
                        maxpsp = peakFreqPPM;
                    }
    }
    /*Pick most downfield side of multiplet*/
    mintpp=maxips*ppsens;
maxpsp=0.0;
    for (i=0; i<numPeaks; i++)
    {
                                    peakIntensity = getPeakIntensity(i);
                                    if(peakIntensity>mintpp)
                                    {
                    peakFreqPPM = getPeakFreqPPM(i);
                    peakFreqHz = getPeakFreqHz(i);
                    if (peakFreqHz >= maxpsh)
                    {
                        maxpsp = peakFreqPPM;
                        maxpsh = peakFreqHz;
                    }
    }
                    }
/*Flips negative to choose most upfield peak of multiplet*/
for (i=0; i<numPeaks; i++)
    {
                                    peakIntensity = getPeakIntensity(i);
                                    if(peakIntensity>=mintpp)
                                    {
                    peakFreqPPM = getPeakFreqPPM(i);
peakppmneg=peakFreqPPM*m;
maxpspneg=maxpsp*m;
                    if (peakppmneg >= maxpspneg)
                    {
                        minpsp = peakFreqPPM;
                    }
    }
                    }
    freePeakList();
/*Uses central frequency for reference*/
ppmdif=maxpsp-minpsp;
cent=minpsp+ppmdif*0.5;
    FETCHPAR("sf",&sf)
    sfn=sf+(cent-ref)*sf/(1e6);
```

```
    STOREPAR("sf",sfn)
}
WRP(extrctv)
}
extrctv=extractno+w;
w++;
}
END
steno++;
w=1;
extrctv=extractno;
RPROCNO(phpno)
SETCURDATA
}
END
QUIT
```

S13.2 Macro for automatically producing difference spectra for each slice

This Bruker automation script is a macro to automatically take the difference of the reference spectrum minus the irradiated spectrum in each slice and store the difference in consecutive process numbers in the irradiated spectra (mulexpno) folder, from which it must be run. Several sequential CSI experiments can be processed with one run of this script:

```
/*Calculates difference spectra and stores in a procno in the off-resonance image dataset*/
/*The on and off-resonance CSI datasets should have processed and the individual */
/*rows extracted using the previous automation script*/
/*This AU is not fully tested and comes without warranty.*/
/*The script works on Bruker Topspin 3.6.2 but has not been tested on other versions*/
/*Use kill command if all goes wrong*/
/*Serena Monaco and Matthew Wallace, 9/2022*/
/*University of East Anglia, matthew.wallace@uea.ac.uk*/
char disk1[32], user1[32], location[128];
int ne=1;
int dif=10;
int phpno=1;
int strpno=42;
int extractno=2;
int extrctv=extractno;
int offno=1;
int np=16;
GETCURDATA
int onno=expno;
strcpy(location,disk);
GETSTRING("Enter location of dataset",location)
GETINT("Enter experiment number of first on-resonance dataset",onno)
GETINT("Enter difference in experiment number between off and on resonance datasets:",dif)
offno=onno+dif;
GETINT("Enter experiment number of first off resonance dataset",offno)
GETINT("Enter number of 2D on/off dataset pairs to process",ne)
GETINT("Enter procno containing 2D data :",phpno)
GETINT("Enter procno containing first extracted row",extractno)
extrctv=extractno;
strpno=extractno+40;
GETINT("Enter procno to store first difference spectrum",strpno)
REXPNO(offno)
RPROCNO(phpno)
SETCURDATA
FETCHPAR1("SI",&np)
GETINT("Enter number of points in CSI dataset (autodetects) :",np)
TIMES(ne)
{
        TIMES2(np)
        {
                                REXPNO(offno)
RPROCNO(extractno)
SETCURDATA
/*have to create a 1D procno to store difference in*/
WRP(strpno)
RPROCNO(strpno)
SETCURDATA
        DATASET(name,offno,strpno,disk,user)
        DATASET2(name,offno,extrctv,disk,user)
        DATASET3(name,onno,extrctv,disk,user)
        STOREPAR("DC",-1.0)
        ADD
        extrctv++;
        strpno++;
        }
        END
        strpno=extractno+40;
        extrctv=extractno;
        offno++;
        onno++;
}
END
QUIT
```

S13.3 Macro for STD factor calculation of each STD NMR difference slice

The Bruker automation script below is a macro allowing automatic STD factor calculation from each slice of the experiment from a comparison of integrals of the off-resonance and difference spectra.

```
/*Calculates STD based on integral*/
/*Create integral file containing each peak of interest (maximum of seven)*/
/*Save this file using wmisc command*/
/*Enter the name below in place of Wstd in: strcpy(text,"Wstd")*/
/*or type/paste name of integral in text box when running this AU script*/
/*Run this AU for an off-resonance dataset which contains the off-resonance*/
/*and difference spectra in separate procnos (as created with separate AU script)*/
/*STD factors (Ioffres-Isat)/Ioffres are calculated for each integral region defined*/
/*and stored in a text file in separate columns*/
/*each line of the text file is a sequential row of the CSI dataset*/
/*This AU is not fully tested and comes without warranty.*/
/*The script works on Bruker Topspin 3.6.2 but has not been tested on other versions*/
/*Use kill command if all goes wrong*/
/*Serena Monaco and Matthew Wallace, 9/2022*/
/*University of East Anglia, matthew.wallace@uea.ac.uk*/
#include <inc/exptUtil>
FILE *fpnt,*fstd;
char dconvdir[256],location[256],dummystr[256],printname[256];
double lppm,rppm,bias,slope,peakIntensity,maxips;
double intgr,ppmdn,ppmup;
int intnum;
double offres1,offres2,offres3,offres4,offres5,offres6,offres7=0;
double diff1,diff2,diff3,diff4,diff5,diff6,diff7=0;
double std1,std2,std3,std4,std5,std6,std7=0;
double pc=0.1;
float min=0;
int linenum=1;
int pkcnt=0;
int offno=expno;
int intnov=3;
int np=15;
int extractno=procno;
int strpno=extractno+40;
int i, numPeaks;
strcpy(location,disk);
strcpy(text,"Wstd");
GETCURDATA
GETSTRING("Enter location of dataset",location)
GETSTRING("Which integral file to use?",text)
GETINT("Enter procno of first off res spectrum:",extractno)
GETINT("Enter procno of first difference spectrum:",strpno)
if ((fstd = fopen(PROCPATH("STD factors.txt"),"wt")) == 0)
   STOPMSG("Cannot create file to store STD factors")
GETINT("Enter number of points in image:",np)
/*Read in integral file*/
TIMES(np)
		{
				RPROCNO(extractno)
				SETCURDATA
RMISC("intrng",text)
STOREPAR("INTSCL",-1.0)
FETCHPAR("CURPRIN",printname)
STOREPAR("CURPRIN","integrals.txt")
LI
STOREPAR("CURPRIN",printname)
linenum=1;
sprintf(dconvdir,"%s/%s/%i/pdata/%i/integrals.txt",location,name,expno,procno);
fpnt=fopen(dconvdir, "r");
fgets(dummystr, sizeof(dummystr), fpnt);
while (fgets(dummystr, sizeof(dummystr), fpnt) != NULL)
{
		/*Need to selectively elimate rows from dconpeaks, then scan for numbers*/
		(void) sscanf(dummystr,"%lf %lf %lf %lf",
						&intnum,&ppmdn,&ppmup,&intgr);

										if(linenum==5)
						{
								offres1=intgr;
						}
						if(linenum==6)
						{
								offres2=intgr;
						}
						if(linenum==7)
						{
								offres3=intgr;
						}
						if(linenum==8)
						{
								offres4=intgr;
						}
						if(linenum==9)
						{
								offres5=intgr;
						}
								if(linenum==10)
						{
								offres6=intgr;
						}
						if(linenum==11)
```

```
                                        {
                                                offres7=intgr;
                                        }
                                        linenum++;
                                        }
   RPROCNO(strpno)
   SETCURDATA
 RMISC("intrng",text)
STOREPAR("INTSCL",-1.0)
FETCHPAR("CURPRIN",printname)
STOREPAR("CURPRIN","integrals.txt")
LI
STOREPAR("CURPRIN",printname)
linenum=1;
sprintf(dconvdir,"%s/%s/%i/pdata/%i/integrals.txt",location,name,expno,procno);
fpnt=fopen(dconvdir, "r");
fgets(dummystr, sizeof(dummystr), fpnt);
while (fgets(dummystr, sizeof(dummystr), fpnt) != NULL)
{
                /*Need to selectively elimate rows from dconpeaks, then scan for numbers*/
                (void) sscanf(dummystr,"%lf %lf %lf %lf",
                                        &intnum,&ppmdn,&ppmup,&intgr);
                                        /*fotr is first, then DMSO, then acetate*/
                                        if(linenum==5)
                                        {
                                                diff1=intgr;
                                        }
                                        if(linenum==6)
                                        {
                                                diff2=intgr;
                                        }
                                        if(linenum==7)
                                        {
                                                diff3=intgr;
                                        }
                                        if(linenum==8)
                                        {
                                                diff4=intgr;
                                        }
                                        if(linenum==9)
                                        {
                                                diff5=intgr;
                                        }
                                                if(linenum==10)
                                        {
                                                diff6=intgr;
                                        }
                                        if(linenum==11)
                                        {
                                                diff7=intgr;
                                        }
                                        linenum++;
                                        }
   std1=diff1/offres1;
   std2=diff2/offres2;
   std3=diff3/offres3;
   std4=diff4/offres4;
   std5=diff5/offres5;
   std6=diff6/offres6;
   std7=diff7/offres7;
   fprintf(fstd,"%f %f %f %f %f %f %f\n",std1,std2,std3,std4,std5,std6,std7);
  extractno++;
  strpno++;
                                        }
   END
   fclose(fstd);
   fclose(fpnt);
QUIT
```

S13.4 Macro for running Imaging STD NMR on Icon

This Bruker automation script is a macro to run Imaging STD NMR on automated instruments run through Icon

```
/*Script for running Imaging Saturation Transfer Difference (STD) NMR experiments under full automation*/
/*Through Bruker IconNMR*/
/*d20 (presaturation time) and cnst20 (on-resonance presaturation frequency) should be*/
/*editable variables that can be changed for each experiment using Icon interface*/
/*Create a parameter set for a quick proton experiment to find frequency of water signal for suppression*/
/*called "H20lock_SS" in script below*/
/*Script will save the d2o, cnst20 number of scans and relaxation delay (d1) entered into Icon, run a quick proton*/
/*then read in the CSI STD parameter set where cnst21 and all other variables are predefined and must be*/
/*edited manually if requried using WPAR command*/
/*STD CSI paramer set is called "STDImage" below*. This is read in and*/
/*d20 and cnst20 set in Icon, and water suppression frequency found experimentally, are stored and the experiment run*/
/*mulexpno for stroing off resonance dataset is set by default as number of experiment in Icon+10*/
/*Make sure this is number clear, or else manually edit this script*/
/*This AU is not fully tested and comes without warranty.*/
/*The script works on Bruker Topspin 3.6.2 but has not been tested on other versions*/
/*Partially based on standard Bruker AUscript, au_watersc*/
/*Serena Monaco and Matthew Wallace, 9/2022*/
/*University of East Anglia, matthew.wallace@uea.ac.uk*/
char path[PATH_MAX];
int noofscans,pscal_save, i, numPeaks;
int offno=1;
double sf, sfo1;
```

```
float d20,cn20,reld;
float peakFreqHz, peakFreqPPM, peakIntensity, maxpsh, maxpsp, maxips;
GETCURDATA
offno=expno+10;
FETCHPAR("d20",&d20)
FETCHPAR("d1",&reld)
FETCHPAR("ns",&noofscans)
FETCHPAR("cnst20",&cn20)
/*Remeber to set peak pikcing regions in this parameter set as required*/
RPAR("H2Olock_SS","all")
ZG
ERRORABORT
EF
ERRORABORT
APK
FETCHPAR("PSCAL",&pscal_save)
STOREPAR("PSCAL",0)
PP
ERRORABORT
  strcpy(path, PROCPATH(0));
    numPeaks = readPeakList(path);

    maxips=0.0;
    maxpsh=0.0;
    for (i=0; i<numPeaks; i++)
    {
                    peakIntensity = getPeakIntensity(i);
                    peakFreqHz = getPeakFreqHz(i);
                    peakFreqPPM = getPeakFreqPPM(i);
                    if (peakIntensity > maxips)
                    {
                        maxips = peakIntensity;
                        maxpsh = peakFreqHz;
                        maxpsp = peakFreqPPM;
                    }
    }
    freePeakList();

    FETCHPAR("SF",&sf);
    sfo1 = sf + maxpsh * 1.0e-6;
    STOREPAR("SFO1",sfo1);
SETCURDATA
RPAR("STDImage","all")
STOREPAR("d20",d20)
STOREPAR("d1",reld)
STOREPAR("ns",noofscans)
STOREPAR("cnst20",cn20)
STOREPAR("SFO1",sfo1)
/*Setup mulexpno for STD experiment by creating a copy of the experiment in offno*/
WRA(offno)
STOREPAR("MULEXPNO1",offno)
ZG
QUIT
```

S14. STD NMR data processing: manual method and automation

STD NMR factors (η_{STD}) are defined as $(I_0\text{-}I_{sat})/I_0$, where I_0 is the signal intensity of the reference spectrum (off-resonance spectrum), and I_{sat} is the peak intensity of the saturated spectrum (on-resonance spectrum), therefore $I_0\text{-}I_{sat}$ is the peak intensity of the difference spectra obtained subtracting the on-resonance from the off-resonance spectrum.

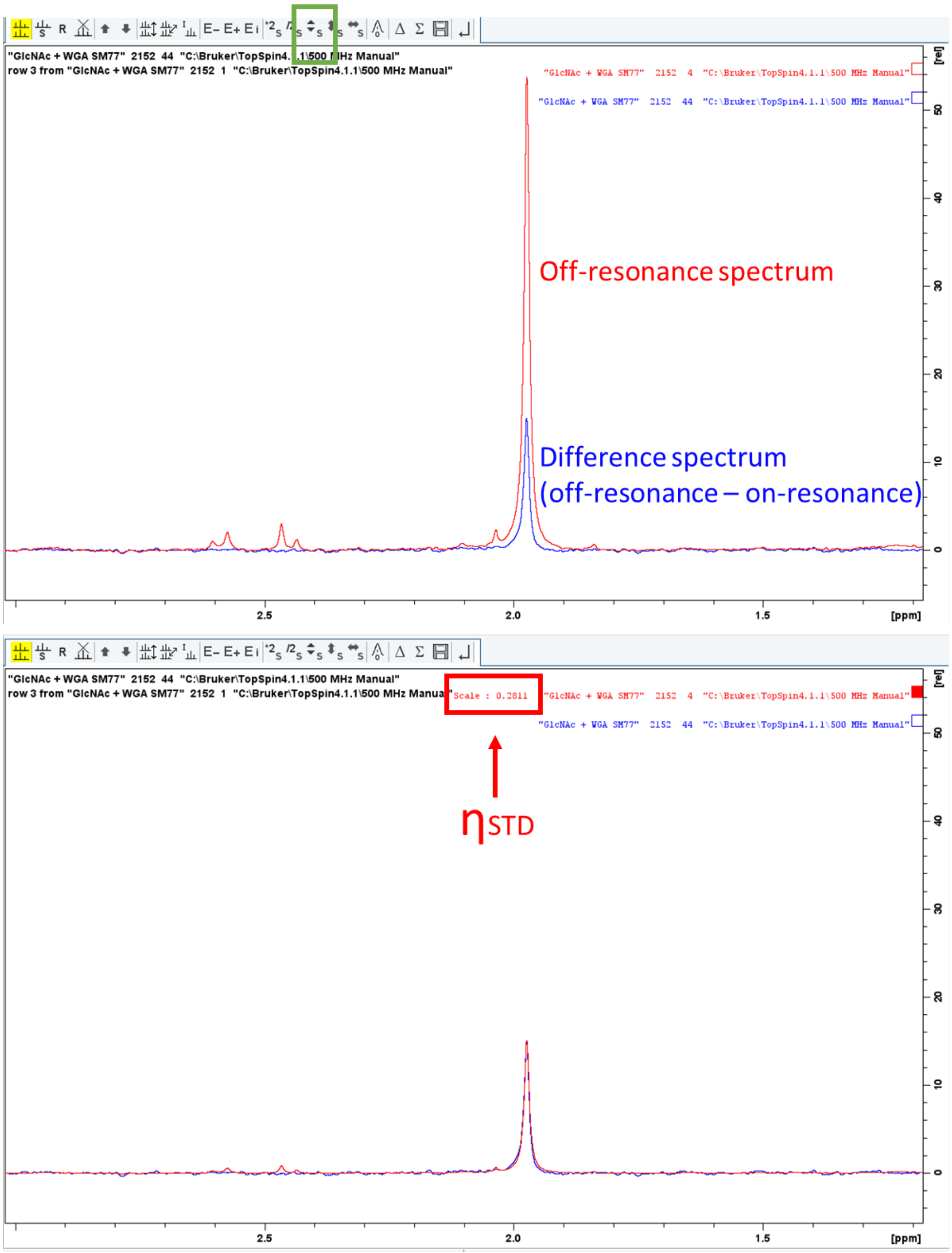

Figure S10. Example of shape-matching procedure to obtain η_{STD} manually. Scaling down is done by using the symbol squared in green in the top screen, and the scaling factor, i.e. η_{STD}, can be read in the top of the bottom screen, circled in red. This procedure is to be repeated for each signal of each spectrum.

The traditional and accurate method to obtain η_{STD} is by "shape-matching" each signal, by manually scaling down the off-resonance spectra until the shape and height match

exactly the signal of the difference spectrum; from the mathematical principles of similarity and scale in geometric transformations, the scaling factor multiplied by 100 corresponds to the η_{STD}(%) value of that signal. Interestingly, for a well isolated and intense singlet, such as the *N*-acetyl's methyl group of GlcNAc we have managed to automate the data processing (based on integration of the resonance, script in section S13.3), obtaining a good match with the manual data processing. The STD-AF build-up curves for this proton at increasing ligand concentration are reported below, and in the following table.

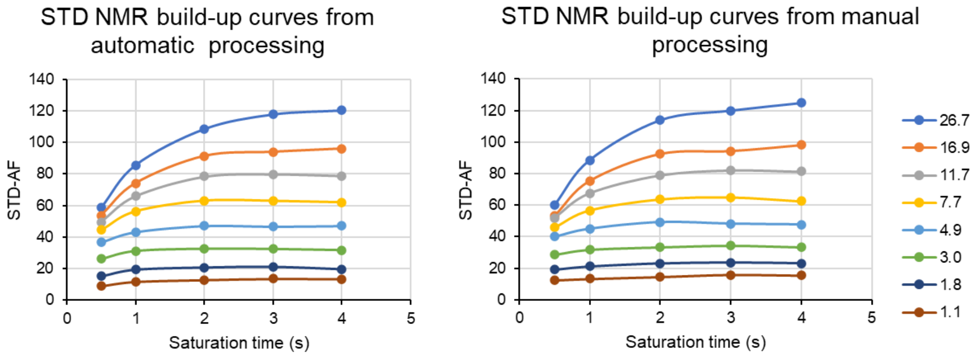

Figure S11. STD-AF build-up curves for the methyl group of the *N*-acetyl moiety of GlcNAc as bound to WGA, coming from automatic data processing on the left, and manual processing on the right. The match between the two curves is very good, showing that the automated integration method works successfully for intense and well isolated singlets.

[GlcNAc]/mM	Processing method	0.5 s	1 s	2 s	3 s	4 s
26.7	Automated	58.67	85.72	108.68	117.92	120.47
	Manual	60.00	88.62	113.85	119.95	124.74
16.9	Automated	53.71	74.11	91.33	93.97	95.97
	Manual	53.07	75.42	92.57	94.38	98.15
11.7	Automated	49.46	65.91	78.41	79.91	78.89
	Manual	51.94	67.59	78.99	82.15	81.35
7.7	Automated	44.46	56.30	62.99	62.88	61.97
	Manual	45.75	56.70	63.75	65.01	62.51
4.9	Automated	36.70	43.24	47.19	46.79	47.22
	Manual	40.04	45.02	49.15	48.20	47.73
3.0	Automated	26.29	31.29	32.68	32.54	31.81
	Manual	28.58	31.82	33.41	34.41	33.42
1.8	Automated	15.32	19.44	20.82	21.25	19.75
	Manual	19.22	21.19	23.02	23.59	23.14
1.1	Automated	9.08	11.73	12.86	13.59	13.47
	Manual	12.46	13.34	14.57	15.91	15.45

Table S15. STD-AFs for the methyl group of the *N*-acetyl group of GlcNAc, at each ligand concentration and increasing saturation times, as obtained automatically and manually.

S15. STD NMR data processing on Mnova 14.3.1 by line fitting

Run this macro (edmac) on Bruker Topspin, then open 2rr file of dataset in Mnova. For off-resonance dataset, the file pulseprogram must be copied over from the on-resonance (executed) dataset if it is not already present.

```
# Sets up a CSI dataset for opening and processing in Mnova
# Sets SI to 32 and 32768, LB 1, phase sensitive in both dimensions (adjust if not appropriate)
#The script works on Bruker Topspin 3.6.2 but has not been tested on other versions
#Matthew Wallace, 1/2023
#University of East Anglia, matthew.wallace@uea.ac.uk
1 SI 16
2 SI 32768
2 LB 3
2 WDW EM
1 WDW SINE
2 PHC1 0
2 PHC0 0
2 PH_mod pk
1 PH_mod pk
#1 PHC1 should be 180*number of gradient points acquired
1 PHC1 2880
XFB
#Now open 2rr file from procno folder into Mnova
```

Setup and run this processing template to phase the spectra and reference to pyrazine (8.577 ppm). Rows 1 and 16 may be distorted as these lie outside the active region of the NMR coil.

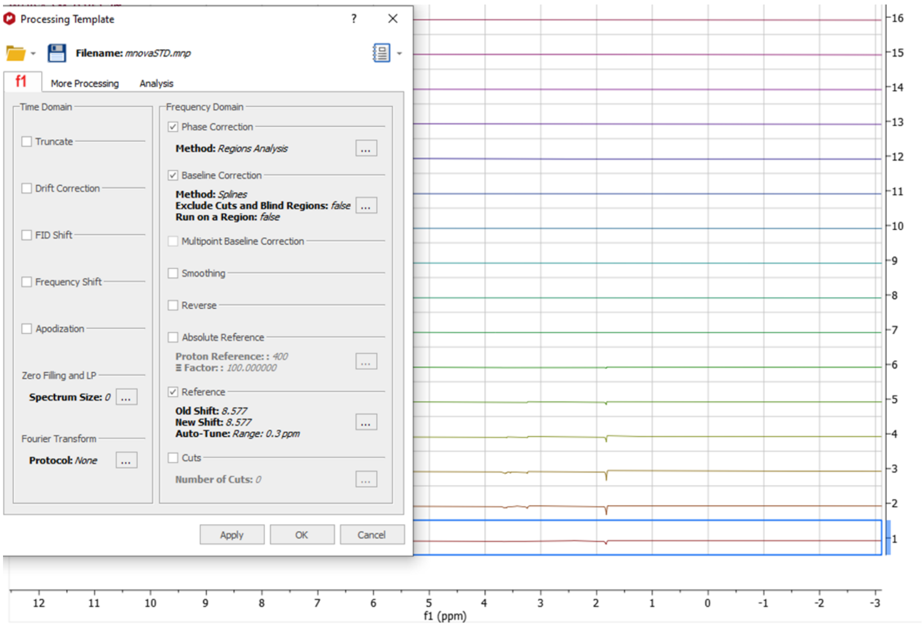

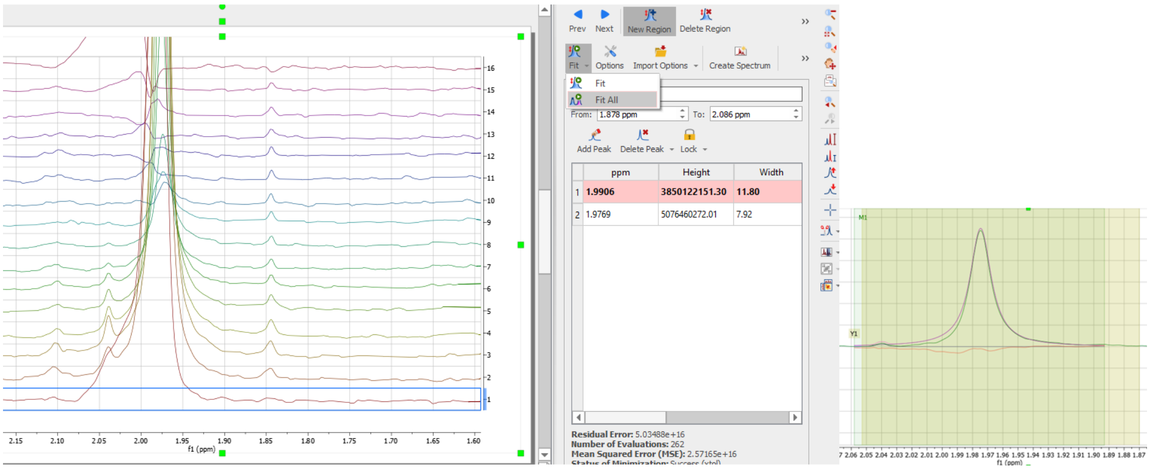

Select the reference (pyrazine) region, then the regions of the ligand, then fit the line regions using the line fitting tool. Save the script below as STDmultiarea and run to create a text file containing the line areas of the reference and ligand areas in subsequent rows. The fitting can be verified by selecting ‘active spectrum’ mode in the stacked menu.

```
/****************************************************************************************************
Matthew Wallace and Serena Monaco, University of East Anglia, 01/2023 (matthew.wallace@uea.ac.uk)
This script will sum the areas of components that are >= rat*area of the largest component found in the fitting region.
The combined area of these components is then placed in the text file.

Based on Mnova script exportFitRegions (Copyright (C) 2014 Mestrelab Research S.L. All rights reserved, part of the Mnova scripting toolkit).
(Authorized users of Mnova Software may use this file freely, but this file is provided AS IS)
with NO WARRANTY OF ANY KIND, INCLUDING THE WARRANTY OF DESIGN, MERCHANTABILITY AND FITNESS
FOR A PARTICULAR PURPOSE.
****************************************************************************************************/
/*globals settings, Dir, FileDialog, File, TextStream, Application, NMRSpectrum, print, Peak, MnUi*/
/*jslint plusplus: true, indent: 4*/
function STDmultiarea() {
"use strict";
function fitRegionToStream(aFitRegion, aFileStream, aNMRPeaks) {
/* aFileStream.writeln("Area");*/
var p, peak, tst, big, comb, rat, nam;
tst=0;
big=0;
comb=0;
rat=0.5;
nam=0;
fitPeaksIds = aFitRegion.peaks;
for (p = 0; p < fitPeaksIds.length; p++) {
peak = new Peak(aNMRPeaks.byId(fitPeaksIds[p]));
tst=peak.integral;
nam=aFitRegion.name;
/*Find biggest peak*/
if(tst>big)
{
big=peak.integral;
}
}
for (p = 0; p < fitPeaksIds.length; p++) {
peak = new Peak(aNMRPeaks.byId(fitPeaksIds[p]));
tst=peak.integral;
if(tst>big*rat)
{
comb=comb+peak.integral;
}
}
aFileStream.write(comb, "\t", nam, "\n");
}
 var fout, sout, spc, peakList, fitRegions, fr, oldCurSpecIndex, i,
dirSettingsKey = "STDmultiarea/LastDir",
saveDir = settings.value(dirSettingsKey, Dir.home()),
dw = Application.mainWindow.activeDocument,
spectra = dw.itemCount("NMR Spectrum"),
specIndex = 0,
fileName = FileDialog.getSaveFileName("ASCII Files (*.txt)", "", saveDir);
 if (!fileName.length) {
return;
}
 fout = new File(fileName);
 settings.setValue(dirSettingsKey, fout.absDirPath);
 if (!fout.open(File.WriteOnly)) {
throw "Impossible to open file";
}
 sout = new TextStream(fout);
 sout.precision = 10;
 while (specIndex < spectra) {
```

```
spc = new NMRSpectrum(dw.item(specIndex, "NMR Spectrum"));
specIndex++;
if (!spc.isValid()) {
throw "Invalid Spectrum";
}
oldCurSpecIndex = spc.curSpecIndex;
for (i = 0; i < spc.specCount; i++) {
spc.curSpecIndex = i;
peakList = spc.peaks();
fitRegions = spc.fitRegions();
print(fitRegions);
for (fr = 0; fr < fitRegions.length; fr++) {
fitRegionToStream(fitRegions[fr], sout, peakList);
}
}
spc.curSpecIndex = oldCurSpecIndex;
 }
 fout.close();
}
if (this.MnUi && MnUi.scripts_nmr) {
MnUi.scripts_nmr.scripts_nmr_ExportASCIIFitRegions = STDmultiarea;
}
```

Files for on- and off-resonance datasets are pasted into spreadsheet which automatically calculates STD factors and ligand concentrations relative to pyrazine.

	A	B	C	D	E	F	G	H	I	J	K	L	M
1	On resonance	2109		Off resonance	2114								Correction factor:
2	67571298.59	M1		105276349.4	M1				Region 1 is pyrazine, Region 2 is Me of GlcNac				Vol of protein solution/
3	222526688.1	M2		249251924	M2	Number of fitting regions:	2		Peak areas				Vol of ligand solution/u
4	243730622.2	M1		238934000.7	M1				On resonance			Off resonance	
5	360777976	M2		360002826.6	M2			Region:	1	2	Norm to Pyr	1	
6	270668853.2	M1		267838464.6	M1			Row					
7	307058372.3	M2		363142956.3	M2			1	67571298.59	2.23E+08	3.29321314	105276349.4	24925
8	308464599.3	M1		306246545.4	M1			2	243730622.2	3.61E+08	1.48023245	238934000.7	3600028
9	212673872.6	M2		271162583.7	M2			3	270668853.2	3.07E+08	1.13444295	267838464.6	3631429
10	335127118.8	M1		329883015.2	M1			4	308464599.3	2.13E+08	0.68945958	306246545.4	2711625
11	129441333.5	M2		185468908.3	M2			5	335127118.8	1.29E+08	0.38624548	329883015.2	1854689
12	352026320.3	M1		348403672.1	M1			6	352026320.3	64757042	0.18395511	348403672.1	1098373
13	64757041.52	M2		109837343.6	M2			7	368255721.6	35021643	0.09510142	367039864.2	5804109
14	368255721.6	M1		367039864.2	M1			8	379691986.5	11651078	0.0306856	380562839.2	290833
15	35021643.39	M2		58041094.93	M2			9	387950422	7030894	0.01812318	389123480.1	1272045
16	379691986.5	M1		380562839.2	M1			10	388914517.2	922195.2	0.0023712	391676425.2	3604464
17	11651077.9	M2		29083320.5	M2			11	390840005.7	1805227	0.00461884	392673866.7	5603310
18	387950422	M1		389123480.1	M1			12	390019677.5	0	0	391578119.9	1168791
19	7030894.133	M2		12720450.99	M2			13	389594913.9	5827852	0.01495875	394261677.6	2797884
20	388914517.2	M1		391676425.2	M1			14	384090096.3	2443031	0.00636057	392431304.6	3261373
21	922195.2013	M2		3604464.962	M2			15	352802097.1	4898350	0.01388413	356854447.2	498206
22	390840005.7	M1		392673866.7	M1			16	281088784.4	33954416	0.12079605	295543180	4520115
23	1805227.496	M2		5603310.989	M2								
24	390019677.5	M1		391578119.9	M1								
25	0	0		1168791.674	M2								
26	389594913.9	M1		394261677.6	M1								
27	5827851.654	M2		2797884.121	M2								
28	384090096.3	M1		392431304.6	M1								
29	2443030.664	M2		3261373.697	M2								

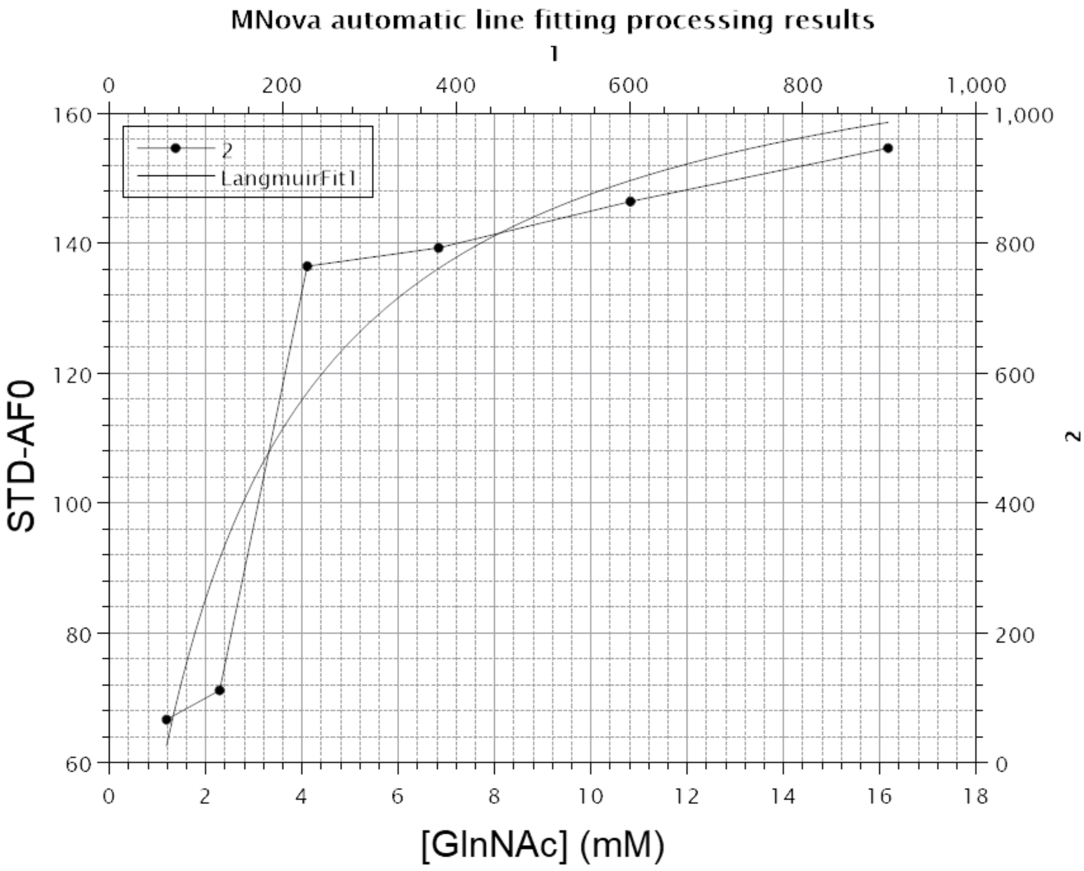

Figure S12. Binding isotherm from STD-AF$_0$ fitting obtained by fitting of data processed by line fitting in Mnova 14, yielding K_D=2.22 ± 0.75 mM.

References

1. Trigo-Mouriño, P., et al., *Probing spatial distribution of alignment by deuterium NMR imaging.* Chemistry–A European Journal, 2013. **19**(22): p. 7013-7019.
2. Adams, R.W., et al., *"Perfecting" WATERGATE: clean proton NMR spectra from aqueous solution.* Chemical Communications, 2013. **49**(4): p. 358-360.
3. Wallace, M., D.J. Adams, and J.A. Iggo, *Titrations without the Additions: The Efficient Determination of p K a Values Using NMR Imaging Techniques.* Analytical chemistry, 2018. **90**(6): p. 4160-4166.
4. Angulo, J., P.M. Enríquez-Navas, and P.M. Nieto, *Ligand–receptor binding affinities from saturation transfer difference (STD) NMR spectroscopy: the binding isotherm of STD initial growth rates.* Chemistry–A European Journal, 2010. **16**(26): p. 7803-7812.
5. Crank, J., *The mathematics of diffusion*. 1979: Oxford university press. 414.
6. Böhme, U. and U. Scheler, *Effective charge of bovine serum albumin determined by electrophoresis NMR.* Chemical Physics Letters, 2007. **435**(4-6): p. 342-345.
